# Shedding Light on Synthetic Autocatalysis: From Conventional Closed‐Shell Chemistries to Overlooked Open‐Shell Occurrences

**DOI:** 10.1002/chem.202502075

**Published:** 2025-10-01

**Authors:** Jaspreet Kaur, Joshua P. Barham

**Affiliations:** ^1^ Fakultät für Chemie und Pharmazie Universität Regensburg Universitätsstraße 31 93053 Regensburg Germany; ^2^ Department of Pure and Applied Chemistry University of Strathclyde 295 Cathedral Street Glasgow G1 1XL UK

**Keywords:** asymmetric amplification, autocatalysis, kinetics, photocatalysis, radicals

## Abstract

Autocatalysis is a kinetic phenomenon where a reaction product catalyzes its own formation, often leading to sigmoidal reaction profiles and exponential rate acceleration. This process plays a fundamental role in biological systems, for example, in chemistry of the origins of life. Although synthetic reactions are not as widely reported as the biological studies, autocatalysis offers a unique mechanism and high efficiency to organic synthesis. This review highlights diverse synthetic examples in various autocatalytic mechanistic paradigms, including well‐studied examples such as the Soai reaction. We also explore emerging examples in radical‐ and photochemistry‐based systems, emphasizing recent advances in auto‐photocatalysis. By exploring examples from different fields, this review aims to highlight the variety and potential of autocatalytic reactions for synthesis. We aim to encourage further research into autocatalysis as a powerful strategy for improving reaction efficiency, revealing new reaction pathways, and inspiring innovative catalytic methodologies.

## Graphical abstract

Why add another catalyst when the product itself holds the power to catalyze its own formation? Autocatalysis in synthetic chemistry enhances reaction efficiency and uncovers novel catalytic behavior across both closed‐shell and open‐shell systems, expanding reactivity and enabling innovative design strategies.

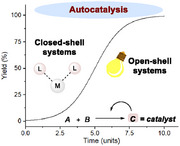

## 1 Introduction

Self‐replication is considered a plausible process in the origins of life. The single‐handedness of core biological compounds — such as sugars and amino acids — suggests that a mechanism might have existed to selectively amplify one enantiomer over the other.^[^ ^1^, ^2^ ^]^ Self‐replication or autocatalysis provides a convincing explanation for this phenomenon, as it enables small initial imbalances to be reinforced and amplified over time. Beyond its relevance to the chiral living world and the origins of life, autocatalysis represents a fascinating kinetic phenomenon studied across multiple scientific disciplines. Recognized by his work on catalysis by the 1909 Nobel Prize, Wilhelm Ostwald had entertained the idea of autocatalysis back in the 1890s.^[^ ^3^, ^4^, ^5^ ^]^ It is broadly defined by its self‐amplifying nature, where a product catalyzes its own formation, accelerating reaction rates in the process. An autocatalytic system is generally associated with an autocatalytic reaction, an autocatalytic cycle, and an autocatalytic set. Without delving too deeply into these terms, they all share one important characteristic — exponential growth — but differ in how this growth is achieved. Hordijk details the definitions of this family of terms in an article titled *Autocatalytic Confusion Clarified*.^[^ ^6^ ^]^

The inherent nature of autocatalysis, which decreases or eliminates the need for external catalysts, makes it a highly sustainable approach in line with the principles of green chemistry. By minimizing waste and improving reaction efficiency, autocatalysis offers a pathway to more environmentally benign chemical synthesis. In synthetic chemistry, autocatalysis in closed‐shell systems has been extensively studied and documented; however, examples involving open‐shell species, such as radicals, remain scarce and underexplored. That is not to say autocatalysis is less relevant in open‐shell systems; rather, it may have been overlooked in a number of existing radical processes. With this review, we aim to highlight this disparity by comparing and contrasting known closed‐ and open‐shell autocatalytic reactions. Our goal is to encourage the community to draw insights from established closed‐shell chemistry to expand the appreciation of autocatalysis within open‐shell systems, particularly its ‘hidden’ operations in existing chemistries and its potential for synthetic applications.

### 1.1 Scope of the Review

This review focuses on chemical reactions that exhibit autocatalysis in both closed‐shell and open‐shell chemistries, with an emphasis on synthetic relevance. We concentrate on examples where the reaction product catalyzes its own formation, excluding biological and template‐based autocatalytic processes, where the template reversibly binds to fragments leading to product formation. To maintain a focused discussion, we also omit cases where the catalyst is a starting material rather than a product,^[^ ^7^, ^8^, ^9^, ^10^, ^11^ ^]^ and examples not directly tied to chemical synthesis, such as photodegradation^[^ ^12^, ^13^, ^14^, ^15^ ^]^ or photodeposition.^[^ ^16^, ^17^, ^18^ ^]^ For a discussion on the different facets of autocatalysis, we encourage readers to refer to these previously published reviews.^[^ ^2^, ^19^, ^20^, ^21^, ^22^, ^23^, ^24^, ^25^, ^26^ ^]^ Unless otherwise stated, quoted yields always correspond to reactions performed without product addition at the start. In some cases, such as asymmetric autocatalysis, where product addition at the start influences enantiomeric excess (ee), yields are shown both without and with product addition at the start.

We begin this review by outlining the general features of autocatalytic mechanisms, followed by a discussion of historical examples to set the context. We also provide a brief citation analysis to highlight the growth of the field and identify which areas are most commonly associated with autocatalysis. The main body of the review is divided into two major sections: closed‐shell and open‐shell systems. The closed‐shell section is further categorized based on how autocatalysis is induced, for example, the product acting as a ligand in metal complexes, product itself acting as a direct catalyst, and other mechanistic distinctions. In contrast, as most reported examples of open‐shell autocatalysis involve photochemistry, this section is organized according to the underlying photochemical mechanisms, including energy transfer, single electron transfer (SET), and related pathways.

### 1.2 Mechanistic Statement of Autocatalysis

**Figure 1:**
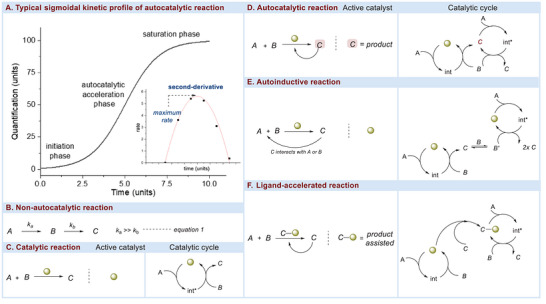
Typical kinetic profile for an autocatalytic process and conceptual catalytic cycles of various autocatalytic processes.

Two characteristic features of autocatalysis are widely recognized (Figure 1A). **Feature 1**) Sigmoidal kinetic reaction profile: Autocatalytic processes exhibit a sigmoidal curve, which can be dissected into: i) a slow *initiation or induction phase* at the onset of the reaction; ii) an *acceleration phase* where the rate of product formation increases to a maximum (an inflection point); and iii) a *saturation phase* where the rate gradually slows as the product molecules (autocatalytic species) outnumber the starting materials. As such, the second derivative of the kinetic profile shows a parabolic shape (Figure 1A, inset). **Feature 2**) Effect of product addition on initial rates: addition of product at the start of the reaction accelerates the reaction and eliminates the induction period.

Diagnosing autocatalysis by interpretation of kinetic profiles (**Feature 1**) should be carried out with caution because not all sigmoidal kinetic profiles would be autocatalytic. As Horváth pointed out in a thorough study on autocatalysis: “*a sigmoidal concentration‐time profile is just a possible but not a unique fingerprint of autocatalysis*”.^[^ ^27^ ^]^ For example, a sigmoidal kinetic curve would be present in a non‐autocatalytic reaction where accumulation of a long‐lived intermediate delays product formation as the product is formed after a noticeable time delay, as in reaction *equation 1* where *k*_a_ ≫ *k*_b_ (Figure 1B). Horváth demonstrated various sigmoidal kinetic models to systematically analyze the presence of autocatalysis in these profiles. Therefore, the unequivocal positive test for autocatalysis is the addition of product (autocatalyst) at the start of the reaction in increasing amounts, to eliminate the induction period and observe accelerated initial reaction rates.

### 1.3 How do Products Participate in Catalytic Cycles?

A typical catalytic cycle includes an exogenous catalyst that accelerates the reaction and works independently of the product or intermediates (Figure 1C). As mentioned earlier, the catalyst in an autocatalytic cycle is the product of the reaction and is the main catalytic pathway toward product formation, even if other background reactions are taking place, such as the mechanism during the initiation phase (Figure 1D). Beyond this classical case, products can influence catalysis in more complex ways (Figure 1E‐F). For example, the product could interact with other reactants to activate them, which can then enter the same catalytic cycle as the initial product formation. Blackmond defined these reactions as *autoinductive reactions*.^[^ ^26^ ^]^ However, Plasson used the term “autoinductive” more specifically to describe cases where the product interacts with the catalyst to enhance its reactivity,^[^ ^28^ ^]^ whereas Blackmond termed such cases “ligand‐accelerated catalysis.”^[^ ^26^ ^]^ Following the strict definition of autocatalysis would mean that reactions where the starting materials are catalysts should not be classified as autocatalytic, though there are reports that classify such reactions as “autocatalytic”.^[^ ^7^, ^8^, ^9^, ^10^, ^11^ ^]^

### 1.4 Historical Examples and Citation Analysis

#### 1.4.1 Formose Reaction

**Figure 2:**
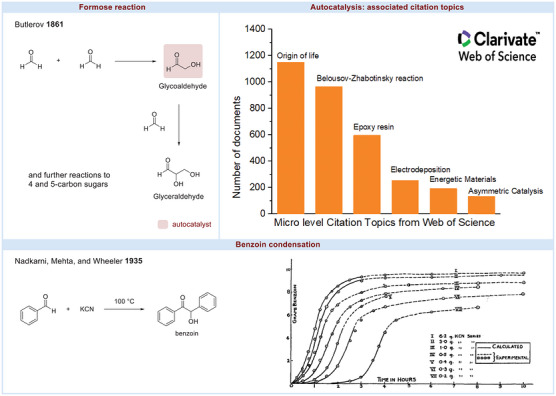
Top, left, formose reaction. Top, right, micro‐level citation topics associated with a Web of Science search for documents containing keywords “autocatalysis” or “autocatalytic” or “asymmetric amplification” or “asymmetric autocatalysis” or “Soai reaction”. https://www.webofscience.com/wos/woscc/summary/91ac72d7‐0912‐4fe9‐8207‐6be037b3fd27‐01493d6feb/relevance/1. Bottom, benzoin condensation in neat benzaldehyde and its corresponding kinetic profile. Kinetic profile is reprinted with permission.^[^ ^31^ ^]^ Copyright © 1935 American Chemical Society.

Earlier than Ostwald's report on autocatalysis, in 1861, Butlerov reported the formation of sugar‐like substances from formaldehyde under mild basic conditions.^[^ ^29^ ^]^ Nearly a century later, Breslow clarified the mechanism of this formose reaction by analyzing its kinetic profile and demonstrating the disappearance of the induction phase when reaction products such as glycoaldehyde, glyceraldehyde, or dihydroxyacetone were introduced at the onset of the reaction, confirming the autocatalytic nature of the process (Figure 2, left).^[^ ^30^ ^]^ The initiation phase encompasses the formation of glycolaldehyde, possibly through direct condensation of two formaldehyde (C1) molecules. Glycolaldehyde can then act as a catalyst as it reacts with additional formaldehyde molecules in a series of aldol condensations and tautomerizations to form higher sugars, such as trioses (C3) and tetroses (C4). The key step is when the aldotetrose (a C4 sugar) forms from the ketotetrose via tautomerization. This aldotetrose—which is the product of an aldol condensation of two glycolaldehyde molecules—can undergo a reverse aldol reaction, which regenerates the original glycolaldehyde and liberates a new one, thereby sustaining the autocatalytic cycle.

#### 1.4.2 Benzoin Condensation

Another historical example of autocatalysis would be the benzoin condensation; a classic example of Umpolung reactivity, which transforms an electrophile into a nucleophile at the same carbon atom. In 1935, Nadkarni, Mehta, and Wheeler reported the kinetic profile of a neat benzoin reaction using pure benzaldehyde and potassium cyanide (KCN).^[^ ^31^ ^]^ Great care was taken to ensure material purity to avoid variable results from impurities. The authors found the reaction proceeds through two different mechanisms: i) a slow, heterogeneous reaction between solid KCN and benzaldehyde, and ii) a fast, homogeneous autocatalytic reaction taking place in the liquid phase. The authors provided key evidence for this autocatalytic behavior by observing a distinct sigmoidal kinetic profile for benzoin formation (Figure 2, bottom). Crucially, while the initial rate of reaction depended on the quantity of KCN, once the autocatalytic phase began, the reaction rate became “almost independent” of the amount of cyanide present. This demonstrated that a small, dissolved trace of KCN was sufficient for the homogeneous reaction. The autocatalytic nature of the reaction was further confirmed by experiments where adding pre‐formed benzoin led to identical behavior as benzoin formed during the reaction.

It is noteworthy that the more popularized benzoin condensations using homogenous organocatalysts — such as thiazolium^[^ ^32^ ^]^ or triazolium^[^ ^33^ ^]^ ions — do not exhibit (sigmoidal) kinetics akin to autocatalysis. Instead, their kinetics are governed by the intrinsic catalytic cycle: thiazolium salts generally show first‐order dependence on benzaldehyde, with adduct formation, deprotonation to the Breslow intermediate, and nucleophilic attack all contributing partially to the overall rate. Triazolium salts display saturation kinetics where the rate‐limiting step (RLS) shifts from adduct formation to deprotonation at high aldehyde concentrations. Isotopic labeling studies further confirm that both deprotonation and enamine re‐protonation are kinetically significant. The high catalytic efficiency in these systems likely prevents any observable contributions of autocatalysis.

#### 1.4.3 Citation Analysis of Autocatalytic Systems

**Figure 3:**
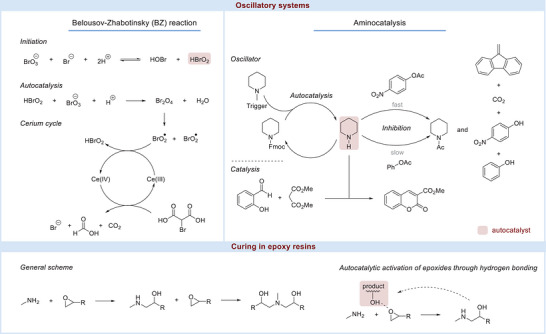
Top, autocatalysis in oscillatory systems such as Belousov‐Zhabotinsky reaction and aminocatalysis. Bottom, autocatalysis in curing of epoxy resins.

A Web of Science search for documents associated with autocatalysis resulted in half the number of references in comparison to the search on SciFinder with the same keywords. Regardless, the Web of Science search (Figure 2, right) shows that autocatalysis is indeed highly associated with the origins of life. The next most frequently associated topic is the Belousov–Zhabotinsky (BZ) reaction, a benchmark in the study of chemical oscillations. The BZ reaction incorporates a complex set of reactions (elementary steps) involving bromine chemistry, oxidation and reduction of the metal catalyst, and various intermediate species (Figure 3, top left).^[^ ^34^, ^35^, ^36^, ^37^ ^]^ Although not all reaction steps are autocatalytic in the BZ reaction, autocatalysis does contribute positively within the feedback loop for the production of bromous acid (HBrO_2_) and oxidation of Ce(III). An application of the BZ reaction was demonstrated in the periodic polymerization of acrylonitrile.^[^ ^38^, ^39^ ^]^ Recently, Harutyunyan and co‐workers reported a synthetic small‐molecule oscillator based on autocatalytic Fmoc (fluorenylmethoxycarbonyl) deprotection, which reinforces the link between autocatalysis and reaction dynamics in chemical systems (Figure 3, top right).^[^ ^40^ ^]^ There are also examples where photochemical processes are incorporated within these oscillatory systems.^[^ ^41^, ^42^ ^]^ Beyond these dynamic systems, autocatalysis is also prevalent in materials science applications, for example, epoxy resin curing, electrodeposition, and energetic materials.

Autocatalysis is a ‘double‐edged sword’ in materials science, offering both advantages and disadvantages. For instance, in thermoset polymers such as epoxy resins, autocatalysis is a positive asset in curing, as the alcohol product formed during the polymerization can activate the epoxide through hydrogen bonding facilitating nucleophilic attack by the amino groups and subsequent ring opening (Figure 3, bottom).^[^ ^43^, ^44^, ^45^, ^46^ ^]^ Similarly, autocatalysis is a synonym for ‘electroless plating’ in the field of materials science, as it serves as a fundamental mechanism in the process. There, the deposited metal accelerates its own formation through the redox reaction while eliminating the need to apply external electric current.^[^ ^47^, ^48^ ^]^ However, autocatalysis can be detrimental in energetic materials because the decomposition products can be highly reactive, decreasing the overall stability of the bulk materials and posing risks during handling and storage.^[^ ^49^, ^50^, ^51^, ^52^ ^]^

## 2 Closed‐Shell Chemistry

### 2.1 Ligand‐Accelerated Autocatalysis

Catalysis involving metal‐based complexes is highly sensitive to the nature of the ligands coordinated to the metal center. In autocatalytic systems, this opens the possibility for the product to enhance or even generate a new catalytic activity, either directly or through the formation of a more active metal‐ligand complex.

**Figure 4:**
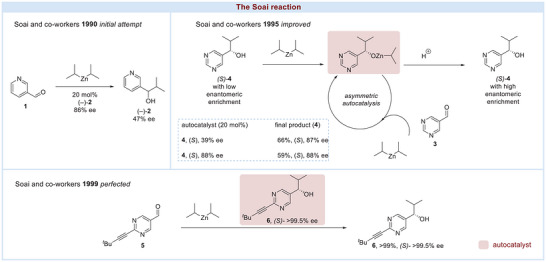
Journey of the autocatalytic Soai reaction for asymmetric amplification.

One of the highlights of autocatalysis is the asymmetric amplification of ee. In 1953, Frank designed a theoretical model to show the possibility of improving ee using autocatalysis.^[^ ^53^ ^]^ The idea illustrated in this mathematical model postulates that one enantiomer can amplify its own formation while suppressing the formation of its mirror image. Soai and co‐workers successfully realized Frank's vision for the amplification of ee in the laboratory decades after this proposal.^[^ ^53^, ^54^, ^55^, ^56^ ^]^ The Soai reaction is regarded as a seminal breakthrough in autocatalysis, although the first few reports from Soai and co‐workers recorded lower ee values of chiral product **2** than of the added chiral catalysts^[^ ^56^, ^57^ ^]^ under asymmetric autocatalytic conditions (Figure 4, top left). It was not until 1995 that the group was successful in achieving true asymmetric autocatalytic amplificatio. This involves alkylation of an aldehyde **3**, where the product **4** forms a chiral zinc alkoxide complex that serves as the catalyst.^[^ ^55^, ^58^ ^]^

As mentioned in Blackmond's review,^[^ ^59^ ^]^ the Soai reaction is the only well‐documented experimental system that fulfills the criteria for Frank's mathematical autocatalytic model. In this reaction, the product catalyzes the reaction of pyrimidyl aldehyde **3** with Zn*^i^* Pr_2_ improving the ee from as little as 0.05% ee upto 88% ee, which is an increase by a factor of 942 (Figure 4, top right). Without extensive mechanistic studies, it was initially proposed that the catalytic step is enantioselective. The chiral catalyst of the reaction is formed from the initial alcohol (product **4**) added, and with each catalytic cycle, more of the chiral catalyst is generated, giving rise to an autocatalytic system. Later, in 1999, Soai and co‐workers published a perfected autocatalytic asymmetric amplification for the formation of *(S)*‐2‐alkynylpyrimidyl alkanol **6**.^[^ ^60^ ^]^ Even after ten successive rounds of asymmetric autocatalysis, *(S)*‐2‐alkynylpyrimidyl alkanol **6** resulted in a 99% yield and 99.5% ee, with no decline in catalytic activity or enantiopurity of the autocatalyst (Figure 4, bottom).

Introduction of the Soai reaction inspired a number of researchers to dig deep into the actual mode of mechanism for enantioselective autocatalysis, the non‐linearity of the reaction, and the transition state. Along with Soai, the mechanistic investigations of Brown, Blackmond, Gridnev, Denmark, and Trapp are noteworthy. Brown and Blackmond thoroughly investigated the mechanism of this reaction through various kinetic studies and revealed that a higher‐order species (e.g. a dimer) could be responsible for autocatalytic asymmetric amplification and not the monomer.^[^ ^54^ ^]^ Interestingly, their kinetic model showed that the active chiral catalyst could be a homochiral dimer, and the heterochiral dimer is an inactive catalyst. Subsequently, other higher‐order species were proposed, such as a tetrameric alkoxide aggregate, a square‐macrocycle‐square (SMS) tetramer, and a macrocyclic dimer.^[^ ^61^, ^62^, ^63^, ^64^, ^65^, ^66^, ^67^, ^68^ ^]^

Another important contribution to the Soai reaction was the work of Denmark and co‐workers,^[^ ^67^ ^]^ who extended this amplifying autocatalytic system from a pyrimidine core to a pyridine, specifically, 5‐(trimethylsilylethynyl) pyridine‐3‐carbaldehyde. Notable observations included the requirement of an isopropyl group for both carbinol (product) and alkylzinc. Any deviation from the isopropyl group resulted in lower catalytic efficiencies. Indications of a catalytically‐active SMS tetramer were shown through a series of NMR studies of various zinc alkoxides showcasing structure‐dependent solution‐state aggregates which displayed similarities to the crystal structure provided by Soai and co‐workers.^[^ ^62^ ^]^ A heteroaromatic core, be it pyridine or pyrimidine, is crucial to the SMS tetramer due to the coordination established by nitrogen with dialkylzinc. Simple phenyl aldehydes showed poor reactivity.

**Figure 5:**
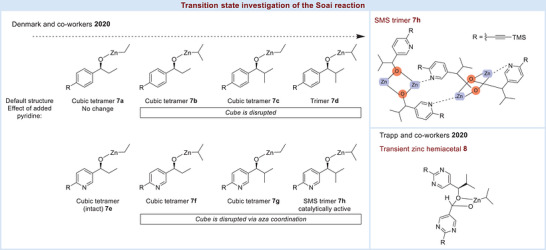
Investigation for catalytically active species for Soai reaction using 5‐(trimethylsilylethynyl) pyridine‐3‐carbaldehyde and corresponding SMS trimer reported by Denmark and co‐workers. Representative transient zinc hemiacetal reported by Trapp and co‐workers.

As shown in Figure 5, NMR studies showed that cubic tetramer **7a** is a preferred constitution for phenyl‐based aromatic rings with ethyl moieties around the group. However, once these ethyl groups are replaced by sterically demanding isopropyl groups (**7b**‐**7d**) and once a pyridine ring is incorporated (**7f**, **7g**), this cubic tetramer is destabilized, leading to the formation of alternative tetrameric aggregates such as the active SMS tetramer **7h**. This SMS tetramer **7h** now contains an aromatic pyridine linker that can allow coordination of the reactant to two unsaturated zinc atoms through two‐point binding. This constrained geometry of the SMS tetramer's structure allows the formation of a homochiral product, favoring alkyl introduction from only one diisopropylzinc‐bound arm.

Recent studies by Trapp and coworkers question the interpretation of the SMS tetramer as the active species, instead providing convincing evidence for dimeric and tetrametric zinc hemiacetals as the active species.^[^ ^69^, ^70^ ^]^ Through in situ high‐resolution mass spectrometry, dynamic HPLC, and detailed kinetic modeling, Trapp's group demonstrated that autocatalysis proceeds via transient zinc hemiacetal complexes **8** — formed between the aldehyde and the chiral alcohol product — and these intermediates enable dual stereocontrol. Their kinetic analysis showed second‐order dependence on aldehyde concentration and first‐order dependence on the alcohol, consistent across different substrate classes (pyridyl and pyrimidyl systems). Their mechanistic model challenges the dominance of the SMS tetramer hypothesis, instead favoring a hemiacetal‐mediated cycle as central to the Soai reaction's enantioselective amplification.

**Figure 6:**
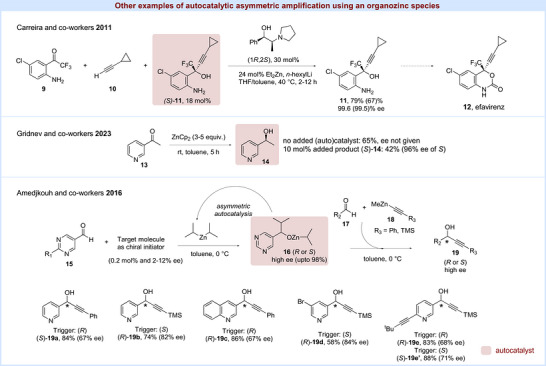
Examples and applications of autocatalytic asymmetric amplification using an organozinc species.

Carreira and co‐workers successfully applied autocatalytic asymmetric amplification using an organozinc species for the asymmetric synthesis of a key intermediate toward the formation of efavirenz **12**, a drug used in the treatment of HIV (Figure 6, top).^[^ ^71^ ^]^ Unlike the above Soai reaction, where aldehydes are the electrophiles, they employed a ketone **9** as an electrophile to access tertiary alcohol **11**. The main advantage of this methodology over existing syntheses of this key intermediate is the catalytic nature of the reaction, where only substoichiometric quantities of the reagents, catalysts, and ligands were needed. There are many differences between this reaction and the well‐known Soai reaction; i) this reaction needed another chiral ligand, which works in synergy with the product (auto)catalyst for asymmetric formation of the alcohol, ii) ethylzinc was used instead of isopropylzinc, iii) the aromatic core of the aldehyde does not contain any heteroatoms. These differences could indicate a different active stereoselective catalyst is present. Notably, this is one of the few applications of a Soai‐type reaction that was also scaled up to meaningful quantities.

Another report from Gridnev extended the scope of Soai‐type reactions by employing dicyclopentylzinc (Figure 6, middle).^[^ ^72^ ^]^ Asymmetric autocatalytic amplification was shown to access *(S)*‐1‐(pyridin‐3‐yl)ethanol **14** from 3‐acetylpyridine **13** despite a decrease in yield. Amedjkouh and colleagues reported distant asymmetric amplification, employing target alkynols **19** with low initial ee values as chiral triggers (Figure 6, bottom). These alkynols **19** activated the Soai autocatalyst **16**, which in turn facilitated their formation with enhanced ee values. Although the scope for aromatic aldehydes was wider than previous reports, the improved ee values were not greater than 90% and fell within the range of 67–82% for five substrates (**19a**‐**19e**).^[^ ^73^ ^]^ Nonetheless, this is an interesting concept to access enantiopure alkynols and could influence future applications of the Soai reaction.

**Figure 7:**
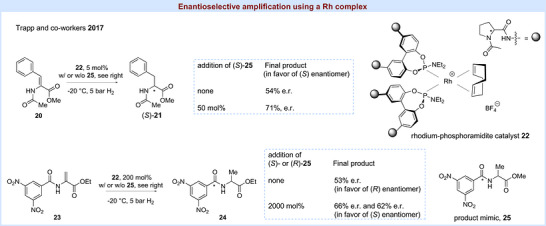
Enantioselective self‐amplification using Rh‐complex.

The literature for asymmetric amplification is saturated with employment of organozinc complexes. Trapp and co‐workers reported a notable exception by employing rhodium‐phosphoramidite catalyst **22** for the enantioselective hydrogenation of dehydroamino acids **20** and **23** (Figure 7).^[^ ^74^ ^]^ They employed presynthesized enantiopure product mimic **25,** which led to immediate increases in enantioselectivity, which supports the role of product in modifying the catalyst to influence enantioselectivity. For the hydrogenation of **20**, addition of enantiopure product mimic **25** increased enantioselectivity to 71% er (in favor of the *(S)*‐enantiomer), showing self‐amplification. This was in comparison to 54% er (in favor of the (*S*)‐enantiomer) when no product mimic was added. For the hydrogenation of **23**, 2000 mol% of *(S)* or *(R)* product mimic **25** was needed, which changed the selectivity in favor of the *(S)*‐enantiomer over the *(R)*‐enantiomer, in comparison to the reaction with no added product mimic. NMR studies confirmed strong and enantioselective non‐covalent binding of the product and catalyst are needed for enantioselectivity amplification.

**Figure 8:**
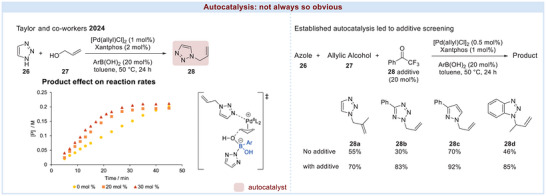
Recognition of autocatalysis prompted additive screening, leading to improved yields. Kinetic profile is reprinted with permission.^[^ ^75^ ^]^ Copyright © 2024 American Chemical Society.

Having explored ligand‐accelerated autocatalysis in asymmetric reactions, we now turn to examples of achiral reactions exhibiting autocatalytic behavior. For example, albeit proceeding in low efficiency, Taylor and co‐workers found autocatalytic behavior of the product **28** of dehydrative couplings of azoles **26** with allylic alcohols **27** under synergistic organoboron/palladium co‐catalysis (Figure 8).^[^ ^75^ ^]^ This study showed how kinetic profiles can be mistaken for *pseudo* zero‐order kinetics, as the reaction curve was rather linear (not sigmoidal) due to the low efficiency of autocatalysis. Autocatalysis could arise as the product **28** coordination to Pd stabilizes a turnover‐determining transition state for ionization. This autocatalytic observation led them to screen various additives which ultimately improved the yields.

**Figure 9:**
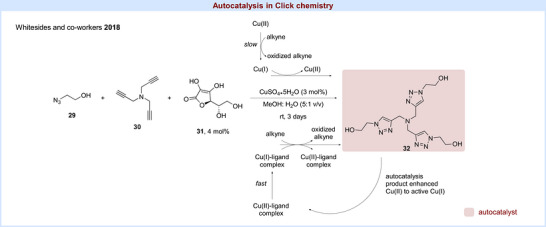
Autocatalysis in click chemistry as the product coordination to Cu enhances its catalytic activity.

Click chemistry, recognized by the 2022 Nobel Prize in Chemistry for the development of copper‐catalyzed azide–alkyne cycloadditions, has also found relevance in the study of autocatalytic systems. Whitesides and co‐workers investigated this transformation through the cycloaddition of tripropargylamine **30** and 2‐azidoethanol **29** (Figure 9).^[^ ^76^ ^]^ The slowest step in the reaction was the formation of the active Cu(I) catalyst via the reduction of Cu(II), which was proposed to proceed through oxidation of the alkyne functionality of the amine **30**. The product, tris‐(hydroxyethyltriazolylmethyl)amine **32**, participated in the reaction by acting as an efficient ligand. It promoted its own formation by first coordinating to Cu(II), facilitating the reduction of Cu(II) to Cu(I), and subsequently enhancing the catalytic activity of Cu(I) during the cycloaddition step. This work revealed a remarkable rate enhancement of over 400 times during the course of the reaction. Similar autocatalytic behavior was observed for the triazoles derived from two other azides; tetraethylene glycol diazide and benzyl azide.

**Figure 10:**
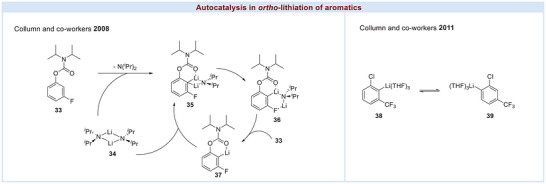
Autocatalysis in *ortho*‐lithiation of aromatic compounds.

In 2008, unusual kinetic observations for the lithiation of 3‐fluorophenyl‐*N*,*N*‐diisopropyl carbamate **33** led Collumn and co‐workers to carry out detailed mechanistic studies (Figure 10).^[^ ^77^ ^]^ Based on kinetic and structural analyses, they demonstrated that this rate behavior during *ortho*‐lithiation arises from both mixed aggregates and autocatalysis. They proposed that an intermediate formed during the reaction acts as a more efficient lithiating agent than the LDA dimer. Specifically, the autocatalytic behavior was attributed to the formation of mixed dimers (LDA–ArLi), with dimers **35** and **36** generated via condensation of aryllithium **37** with LDA dimer **34**.

A similar theme of autocatalysis was seen in further studies from the same group. They observed the effect of regioisomers on autocatalysis during *ortho*‐lithiation of 1‐chloro‐3‐(trifluoromethyl)benzene **38**.^[^ ^78^ ^]^ For example, autocatalytic activity of regioisomer **38** improved six times versus **39**, and an equilibration pathway could convert **39** to **38**. The effect of autocatalysis for *ortho*‐lithiation also switches the lithiation to monomer‐based metalation from the usual dimer‐based metalation for uncatalyzed (initiation step) reactions. Lithiation of 2‐fluoropyridines also proceeded by an autocatalytic mechanism.^[^ ^79^ ^]^ Unlike the above example, *ortho*‐lithiation of 1,4‐difluorobenzene showed weak autocatalysis^[^ ^80^ ^]^ and autocatalysis was deemed unimportant for 1,4‐bis(trifluoromethyl)benzene.^[^ ^81^ ^]^ This could be because the reaction was only marginally catalyzed by the aryllithium intermediate. These species did not significantly enhance the metalation, unlike in other systems where autocatalysis by intermediates was more pronounced.

**Figure 11:**
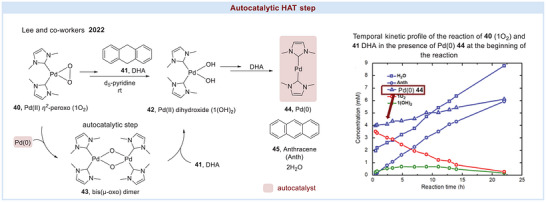
Oxidation addition of Pd(II) product to Pd(II) η^2^‐peroxo complex leads to an autocatalytic HAT step. Kinetic profile is reprinted with permission.^[^ ^82^ ^]^ Copyright © 2022 American Chemical Society. The kinetic profile shows the concentration of different species of the reaction over the course of the reaction.

Lee and co‐workers investigated the oxygen reduction reaction using 9,10‐dihydroanthracene **41** (DHA) as a hydrogen donor under Pd‐catalyzed conditions (Figure 11).^[^ ^82^ ^]^ The reaction proceeded via a Pd(II) η^2^‐peroxo complex **40**, which oxidized DHA **41** to yield anthracene **45**, water, and a Pd(0) product **44**. Addition of the Pd(0) **44** product species eliminated the induction period, revealing an autocatalytic mechanism (Figure 11, right). This arises as product Pd(0) **44** undergoes oxidative addition into the O─O bond of Pd(II) η^2^‐peroxo complex **40** to form a transient bis(μ‐oxo) Pd(II) dimer **43**. DFT calculations showed that the bis(μ‐oxo) Pd(II) dimer **43** has a lower barrier for hydrogen atom transfer (HAT) with DHA **41**. Although this report proposed a HAT step, which implies the presence of radical species, no radicals were detected by EPR.

### 2.2 Product as Autocatalyst

**Figure 12:**
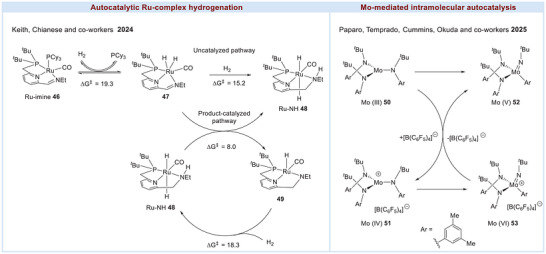
Metal‐complex products acting as direct autocatalysts.

Keith, Chianese, and co‐workers demonstrated autocatalysis in the operation of a ruthenium‐PNN‐pincer hydrogenation catalyst. They discovered that the Ru‐NH complex **48** itself facilitates the hydrogenation of the reactant Ru‐imine **46** (Figure 12, left).^[^ ^83^ ^]^ This was confirmed with kinetic experiments under varying concentrations of PCy_3_, hydrogen pressure, and Ru‐imine **46**. Computational DFT studies confirmed a low‐barrier pathway in which Ru‐NH **48** transfers hydrogen to Ru‐imine **46** intermediates.

Paparo, Temprado, Cummins, Okuda, and co‐workers explored an autocatalytic oxidation mechanism in a molybdenum(III) tris(anilide) **50** system, showcasing a pathway for intramolecular oxidative addition (Figure 12, right).^[^ ^84^ ^]^ Oxidation of Mo(III) complex **50** initiates an intramolecular oxidative addition across the N─C*_ipso_* bond of an anilide ligand, yielding a Mo(VI) imido/aryl complex **53**. This Mo(VI) product **53** acts as an autocatalyst, promoting further conversion of the Mo(III) precursor **50** through a redox‐mediated pathway. The Mo(V) species **52** obtained via reduction of the product is thermodynamically favored but inaccessible directly due to a high kinetic barrier, which is circumvented through the autocatalytic cycle.

**Figure 13:**
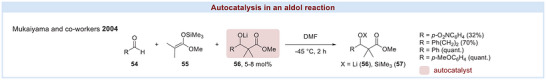
Autocatalysis in Aldol reaction where the product acted as a Lewis base catalyst.

Mukaiyama and co‐workers reported an aldol reaction where they used catalytic amounts (5 mol%) of the desired aldolate product **56** at the start of the reaction as a Lewis base (auto)catalyst (Figure 13).^[^ ^85^ ^]^ This allowed them to avoid using any external alkoxide anion as a catalyst and overcame the induction phase. The aldolate first coordinates to the Si atom of trimethylsilyl enolate. Subsequent coordination of the DMF solvent generates a highly nucleophilic, hexa‐coordinate hypervalent silicate species, which can then attack the aldehyde **54**. By implementing autocatalysis, the authors obtained yields from 90% to quantitative for aromatic aldehydes with electron‐donating groups. However, yields suffered for aromatic aldehydes bearing electron‐withdrawing groups and for aliphatic aldehydes.

**Figure 14:**
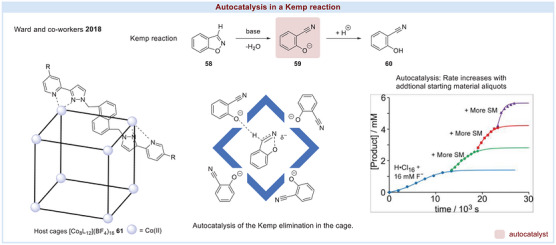
Autocatalysis in the Kemp elimination in a host cage. Kinetic profile is reprinted with permission,^[^ ^86^ ^]^ licensed under CC BY 4.0. Copyright © 2022 American Chemical Society.

Another interesting example of autocatalysis was presented by Ward and co‐workers for catalysis within a cationic metal–ligand coordination cage **61** (Figure 14).^[^ ^86^ ^]^ They used [Co_8_L_12_](BF_4_)_16_ host cage **61**, which contained cobalt(II) ions with a naphthalene spacer and two chelating units (pyrazole and pyridine). They analyzed the effect of accumulated hydroxide anions **59** around the cationic cage and reported an increased rate for the Kemp reaction of bound benzisoxazole **58**. In contrast, other anions such as halide anions inhibited (or slowed down) the reaction, but this allowed another mechanism to function, namely autocatalysis. The minute quantities of product, 2‐cyanophenolate **59**, accumulated while displacing unreactive halide anions to act as the base. It is noteworthy that the autocatalytic Kemp elimination is only observable through kinetics when halide anions are present because the normal, more rapid reaction pathway involving hydroxide ions is inhibited. The core issue is that a hydroxide ion is the typical base, and its reaction with benzisoxazole is so fast that it outcompetes any other potential reaction pathway.

**Figure 15:**
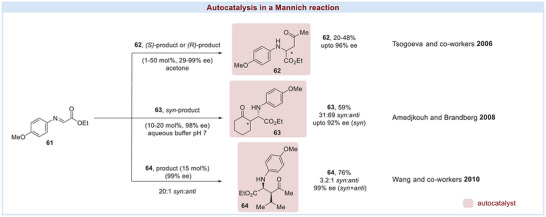
Asymmetric autocatalysis in the Mannich reaction under various conditions.

The 2021 Nobel Prize in Chemistry is a testament to the widespread uptake of asymmetric organocatalysis by synthetic chemists. Thus, combining organocatalysis with autocatalytic chirality amplification would be expected to offer a powerful synergy for asymmetric synthesis. Accordingly, Mannich‐type reactions were investigated as a platform for combining organocatalysis with asymmetric autocatalysis, with a focus on controlling non‐covalent interactions to induce enantioselectivity (Figure 15, top). In 2006, Tsogoeva and co‐workers reported a seminal example of asymmetric (organo)autocatalysis by using the chiral product **62** as a catalyst.^[^ ^87^ ^]^ Despite requiring several days to reach yields of only 20–48%, the reaction of acetone with *N*‐protected α‐imino ethyl glyoxylate **61** exhibited a remarkable increase in ee upon addition of the chiral product **62** (from 0.5–9.5% to 85‐96%), with the extent of enhancement depending on both the amount and the enantiopurity of the product added. The final product **62** retained the same absolute configuration as the added catalyst but exhibited lower enantiomeric purity than the initially added (organo)autocatalyst, highlighting that chirality amplification via autocatalysis is less efficient than in Soai‐type reactions.

Later, Amedjkouh and Brandberg demonstrated the (auto)catalytic activity of the (2*S*,10*S*)‐product **63**. Its addition in 10 mol% led to good yields across all tested solvents (Figure 15, middle); however, the enantioselectivity of the final product **63** was influenced by the choice of solvent.^[^ ^88^ ^]^ For example, use of CDCl_3_ resulted in racemic product (60%), whereas DMF provided a lower yield of product **63** (38%) in an *anti:syn* ratio of 37:63 and in 60% ee. Under aqueous buffered conditions at pH 7, the reaction gave a higher yield of 59% and slightly favored the *syn* isomer with a diastereomeric ratio of 31:69, and in 92% ee. However, consistent with observations by Tsogoeva and coworkers, no noticeable enantiomeric amplification was observed, as the product displayed a lower ee than the added (auto)catalyst.

Wang and coworkers employed an autocatalyst mimic for the Mannich reaction instead of the exact product as the autocatalyst to understand reactivity and stereoselectivity imposed by the catalyst (Figure 15, bottom).^[^ ^89^ ^]^ They showed notable changes occurred to the enantiomeric and diastereomeric purities of autocatalysts after the reaction. Without an autocatalyst mimic, these changes would not be possible to detect. Unlike previous literature, they were able to preserve the enantiomeric purity of the autocatalyst (99% ee) when isovaleraldehyde and imine **61** were reacted using the autocatalyst (product **64** and not a mimic). A product yield of 76% resulted, with a 20:1 *syn:anti* diastereomeric ratio. However, the final diastereomeric ratio eroded to 3.2:1 *syn:anti*, likely due to epimerization of the labile Mannich products at the α‐position during the course of the reaction.

**Figure 16:**
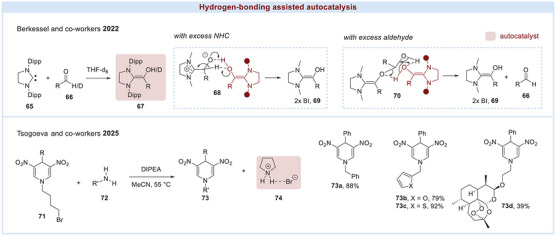
Top, autocatalysis for the formation of the Breslow intermediate. Bottom, autocatalysis in a catalyst‐free transamination metathesis.

Berkessel and coworkers uncovered the autocatalytic behavior of the Breslow intermediate **67** (BI) for its own formation from *N*‐heterocyclic carbenes **65** (NHCs) and aldehydes **66** under aprotic conditions (Figure 16, top).^[^ ^90^ ^]^ Regardless of which of the two reactants was used in excess, BI **67** was found to autocatalyze its own formation. When the NHC was used in excess, BI **69** assisted the otherwise unfavorable 1,2‐C‐to‐O proton shift in the zwitterionic primary adduct **68**, proposed to be facilitated by hydrogen bonding between the NHC **65** and BI **69**. With excess aldehyde, a hemiacetal **70** was first formed, which required an intramolecular H‐shift via a 4‐membered TS to result in BI **69**. BI also autocatalyzed this step through a 1,3‐hydrogen shift via a six‐membered TS.

Building on this concept of hydrogen bonding assisted autocatalysis, Tsogoeva and coworkers recently reported an example of a catalyst‐free transamination metathesis reaction of *N*‐bromobutyl‐substituted 1,4‐dihydropyridines **71** (Figure 16, bottom).^[^ ^91^ ^]^ In their system, the in situ‐formed pyrrolidinium salt **74** acted as a hydrogen bond donor, directly facilitating the multistep reaction cascade without requiring any external catalyst or prior chemical modification. This stabilized reactive intermediate lowered the activation barriers. The reaction exhibited a broad substrate scope, tolerating diverse primary and secondary amines bearing aliphatic, aromatic, and heterocyclic groups (**73a**‐**73d**). It also enabled the synthesis of complex structures, including artemisinin‐based and PROTAC derivatives.

**Figure 17:**
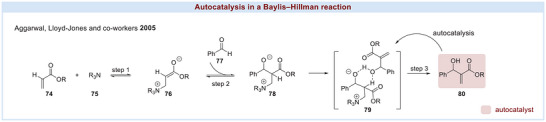
Autocatalysis in Baylis–Hillman reactions.

Aggarwal, Lloyd‐Jones, and coworkers considered autocatalysis might underpin the Baylis–Hillman reaction in aprotic solvents with no external protic source (Figure 17).^[^ ^92^ ^]^ Herein, autocatalysis arises because the product **80** acts as a hydrogen bond donor that promotes a six‐membered TS (**79**) for a proton‐transfer event within the zwitterionic alkoxide, delivering amine elimination via an E1cB‐like pathway (step 3). This is evidenced by i) the loss of the induction period when catalytic amounts of product **80** or methanol are added and ii) kinetic isotope effects showing that proton transfer is the RLS at the start of the reaction and as product accumulates, the RLS switches to a reversible C─C bond‐forming step (step 2). Enantioselectivity is therefore determined at the elimination/proton‐transfer stage. Among the diastereomeric alkoxide adducts generated reversibly, only the one with a properly positioned H‐bond donor undergoes fast, selective proton transfer. The others revert to starting materials (resulting in an enantioenriched product). Moreover, aprotic solvents may be crucial to achieving high ee, whereas competitive non‐selective autocatalysis can erode enantioselectivity.

**Figure 18:**
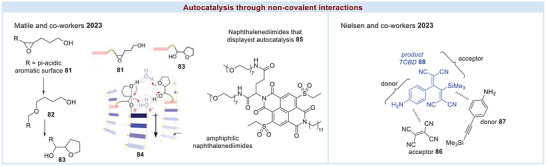
Autocatalysis through non‐covalent interactions. Left, autocatalysis in epoxide‐opening cycloetherifications, reprinted with permission,^[^ ^93^ ^]^ licensed under CC BY 4.0. Copyright © 2023 American Chemical Society. Right, autocatalysis in [2 + 2] cycloaddition retroelectrocyclization reaction.

In a different chemical context, Matile and coworkers discovered that amphiphilic naphthalene diimide micelles **81** can harness anion–π catalysis to autocatalytically promote epoxide‐openings. Subsequent cyclizations give five‐membered ethers (from **81** to **83**); enabled by supramolecular organization within the micellar framework **84** (Figure 18, left).^[^ ^93^ ^]^ While even the least π‐acidic micelles displayed superior performance compared to conventional micellar systems, only the most π‐acidic assemblies exhibited clear autocatalytic kinetics in epoxide‐opening cycloetherifications. Substrate **81** showed a five‐fold enhancement in rate when transitioning from its monomeric nature in THF to its micellar (autocatalytic) nature in water. This effect was rationalized by a transition state in which substrate and product colocalize deep within the micellar core of a polarizable π‐stack, aided by two sandwiched water molecules. The dependence of autocatalysis on substrate hydrophobicity—maximal for **81** and strongly attenuated for less hydrophobic analogues—highlights the critical role of micellar organization and confined space to enable emergent autocatalytic behavior in aqueous media.

Nielsen and coworkers reported autocatalysis in the [2 + 2] cycloaddition‐retroelectrocyclization (CA‐RE) reaction between electron‐rich alkynes **87** and tetracyanoethylene **86** to yield 1,1,4,4‐tetracyanobutadienes **88** (TCBDs) (Figure 18, right).^[^ ^94^ ^]^ They observed that the presence of the TCBD product **88** notably accelerates the reaction. Mechanistically, this effect is attributed to the formation of ternary charge‐transfer complexes involving the product and the reactants, which facilitate the alignment and proximity of the reactive species. Autocatalysis in the CA‐RE reaction through non‐covalent interactions was further corroborated by the work of Lemiègre and Trolez.^[^ ^95^ ^]^

1,1′‐Thiocarbonyldiimidazole (TCDI) is often used as thiocarbonyl transfer agent, resulting in imidazole as one of the by‐products. Paul‐Gorsline and coworkers found imidazole functioned as an autocatalyst when TCDI was employed for the formation of a mixed thiourea and an aryl isothiocyanate.^[^ ^96^, ^97^ ^]^ Duez, Hanusek, Roithová, and coworkers established the autocatalytic role of polysulfide intermediates as they act as internal thiophiles in the Eschenmoser coupling reaction of 3‐bromooxindole and thiobenzamide.^[^ ^98^ ^]^

**Figure 19:**
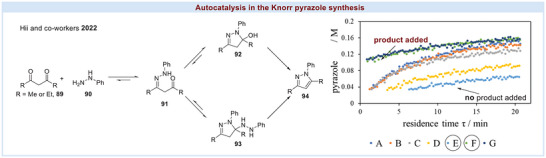
Autocatalysis in the Knorr reaction. Kinetic profile is reprinted with permission,^[^ ^99^ ^]^ licensed under CC BY 3.0. Copyright © 2022 Royal Society of Chemistry.

Autocatalysis was also uncovered in the Knorr pyrazole synthesis from phenyl hydrazine **90** and 1,3‐diketones **89** (Figure 19).^[^ ^99^ ^]^ It played a crucial role in facilitating the final step of the reaction mechanism, namely, the dehydration and aromatization of intermediate species **92** and **93** into the pyrazole product **94**. The kinetic profiles from the ‘different excess’ experiments (Figure 19, experiments A–C) revealed that the reaction's kinetics were complex. Specifically, experiments B and C, which involved varying the initial concentration of phenyl hydrazine **90** and diketone **89**, respectively, were not superimposable. This indicates that changing the concentration of each reactant has a different effect on the reaction rate. The kinetic experiments using transient flow techniques and microkinetic modeling further confirmed that the key reaction steps are effectively inactive in the absence of the pyrazole product **94**. However, they proceed rapidly when catalyzed either by the pyrazole product **94** or by the starting diketone **89**. Elsewhere, the addition of water had no effect on the reaction rate (Figure 19, experiments F–G).

### 2.3 Other Examples

**Figure 20:**
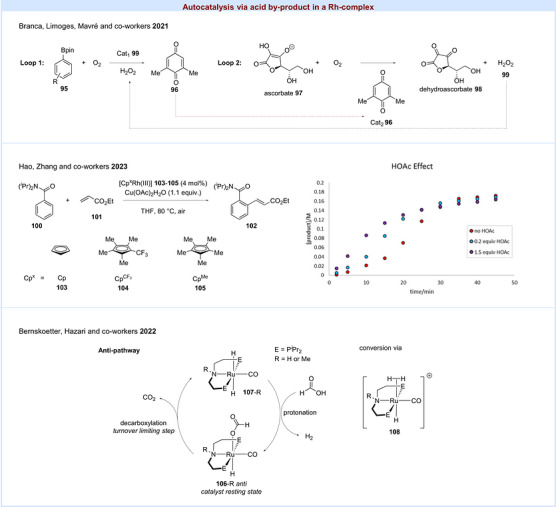
Top, autocatalysis in cross catalysis. Middle, autocatalysis through acid by‐products in C─H olefinations. Kinetic profile reprinted with permission.^[^ ^111^ ^]^ Copyright © 2023 American Chemical Society. Bottom, autocatalysis in CO_2_ hydrogenation using Ru‐based metal complexes.

Small molecules such as acids, water, methanol, and hydrogen peroxide can act as autocatalysts in reactions.^[^ ^100^, ^101^, ^102^ ^]^ Recent examples of acid autocatalysis include the hydrolysis of acetic anhydride to acetic acid,^[^ ^103^ ^]^ hydroiodic acid‐promoted autocatalytic polymer degradation,^[^ ^104^ ^]^ and hydrochloric acid's role in the dechlorination of polyvinyl chloride.^[^ ^105^ ^]^ Water has been shown to act as an autocatalyst in methanol oxidation^[^ ^106^ ^]^ and CO_2_ hydrogenation.^[^ ^107^ ^]^ Hydrogen peroxide (H_2_O_2_) functioned as an autocatalyst in one of the steps of tartaric acid oxidation by Fe(II) sulfate and oxygen.^[^ ^108^ ^]^ An interesting organic autocatalytic reaction network involving cross‐catalysis between H_2_O_2_ **99** and benzoquinone **96** was also reported (Figure 20, top). In that system, the deprotection of an arylboronic ester generates 2,6‐dimethyl‐*para*‐benzoquinone **96**, which catalyzes H_2_O_2_ formation through redox cycling with ascorbate **97**.^[^ ^109^ ^]^ Additionally, the autocatalytic transformation of phosphorylated α‐hydroxybenzyl phosphonates with dialkyl phosphites is initiated by trace water, while the methanol formed acts as an autocatalyst by facilitating proton transfer through coordination in the transition state.^[^ ^110^ ^]^

Another example of autocatalysis involving an acid by‐product was reported by Hao, Zhang, and coworkers in Rh(III)‐catalyzed C─H olefination (Figure 20, middle).^[^ ^111^ ^]^ While examining the steric and electronic effects on the rate‐determining C─H activation step, they found that only the simple [CpRh(III)] complex **103** — with the lowest steric hindrance — exhibited sigmoidal kinetics. The addition of HOAc eliminated the induction period, suggesting that the acid promotes conversion of an off‐cycle Rh species into the active catalyst.

Bernskoetter, Hazari, and coworkers investigated CO_2_ hydrogenation using Ru‐based metal complexes. They identified that the formate product, [H‐DBU][formate], where DBU is 1,5‐diazabicyclo(4.3.0)non‐5‐ene, likely takes part in its own formation by acting as a weak Lewis acid (Figure 20, bottom).^[^ ^112^ ^]^ Its presence facilitated the turnover‐limiting dissociation of the coordinated formate ligand from the ruthenium complex **106**, hence accelerating regeneration of the active hydride species **107**. The addition of [HDBU][formate] at the start of the reaction markedly increased the rate, highlighting its role in promoting further product formation. They suggested that such autocatalysis is probably present in other CO_2_ hydrogenation systems that use amine bases.

## 3 Open‐Shell Chemistry

**Figure 21:**
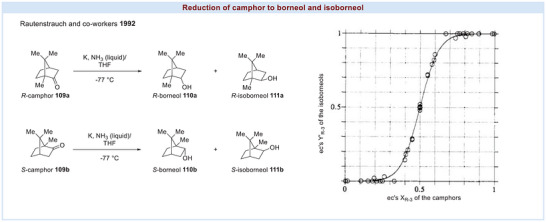
Reduction of camphor using potassium in liquid ammonia, kinetic profile is reprinted with permission^[^ ^113^ ^]^ Copyright © 1992 American Chemical Society.

We now turn our attention to autocatalysis in open‐shell reactivities to include examples that go through radical intermediates. A disparity in the studies of autocatalysis within closed‐ versus open‐shell chemistry is stark. Unlike the widespread attention given to the closed‐shell Soai reaction (1995), the 1992 report by Rautenstrauch and coworkers largely went unnoticed.^[^ ^113^ ^]^ They reported the reduction of camphor using potassium in liquid ammonia (in ammonia/THF at −77 °C) (Figure 21). The enantiomeric composition (ec) for the minor isoborneol product (**111**) was strongly amplified relative to the starting substrate's ec, which the authors explained using the double Horeau duplication mechanism and non‐linear kinetics. However, this behavior was not seen for the formation of the major borneol product (**110**). As much as the kinetic model supported the autocatalytic nature of the formation of the minor product (sigmoidal profile, Figure 21, right), this conclusion is incomplete without the reaction rate information upon the addition of the product. A close inspection of the literature citing this publication revealed that most of the citations are not related to autocatalysis.^[^ ^114^, ^115^, ^116^, ^117^ ^]^

**Figure 22:**
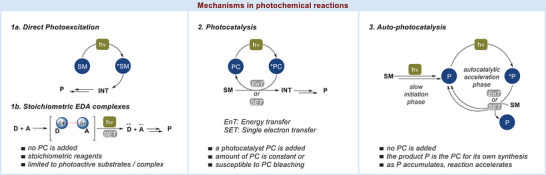
Photochemical pathways. Photochemical mechanisms are adjusted and reprinted with permission.^[^ ^119^ ^]^ Copyright © 2025 Wiley‑VCH GmbH. CC BY 4.0.

Since most of the examples that contain radicals and autocatalysis are linked to the use of photons, we focus specifically on synthetic photochemical examples. Synthetic photochemistry has advanced remarkably since Ciamician's vision of using light to drive chemical reactions in 1912.^[^ ^118^ ^]^ Its development from a conceptual idea to real‐world applications can be attributed in part to a myriad of studies that allowed in‐depth understanding of how light and matter interact. Most photochemical processes go through one of the following mechanisms (Figure 22):

1. Photoactive substrates or photoactive complexes directly absorb light to generate reactive intermediates.
2. Photocatalytic reactions whereby a photocatalyst is needed to harness light energy to either carry reactions via energy transfer or SET.

**Figure 23:**
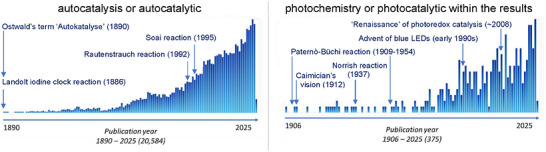
Histogram of the number of publications over the last ∼130 years mentioning “autocatalysis” in general, versus specifically in “photochemical reactions.” Timestamps are given for key events/named reactions in the autocatalysis and photochemical literature. Relative trends are shown without a y‐axis for the number of publications, the total number of publications is given on the x‐axis.

Another mechanism (Figure 22, 3), auto‐photocatalysis, possesses intermediary characteristics of the abovementioned pathways, whereby no external photocatalyst is needed at the start of the reaction, but as the product accumulates the reaction accelerates. This can take place when the product has sufficient photophysical and/or redox properties akin to a photocatalyst. Unlike the identification/development of autocatalytic systems in biology and closed‐shell chemical reactions, autocatalysis in synthetic photochemistry is uncommon/rarely identified and requires more studies to unearth its potential or, its ‘hidden role’ in many (existing) reactions. As per a SciFinder search for the terms “autocatalysis” or “autocatalytic” in publications over the last ∼130 years (∼20.5k hits), a further filtration of the search to show photochemistry‐related publications decreased the hits to only ∼2% (375 hits), showcasing the limited literature available for autocatalysis in photochemistry (Figure 23).

### 3.1 Photoinduced Energy Transfer

**Figure 24:**
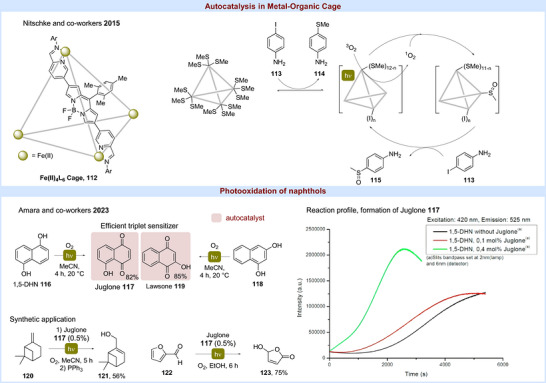
Top, metal‐organic cages for photosensitization to form singlet oxygen. Bottom, autocatalytic photooxidation of naphthols and subsequent applications. Kinetic profile is reprinted with permission^[^ ^123^ ^]^ Copyright © 2023 American Chemical Society.

One of the earlier reports mentioning autocatalysis in a photoinduced system was by Gupta and coworkers in 1976 for photodynamic oxidation of iodide ions (I^−^) to spectroscopically detected I^3−^, using anthracene sulphonates as photosensitizers.^[^ ^120^ ^]^ Photoinduced autocatalytic energy transfer was reported for the enhancement of a visual colorimetric assay.^[^ ^121^ ^]^ In a supramolecular system, Nitschke and coworkers combined the features of self‐assembly and photochemistry in metal‐organic Fe(II)_4_L_6_ (**112**) (Figure 24, top).^[^ ^122^ ^]^ These cages play a dual role as both photosensitizers and substrates: upon irradiation they generate singlet oxygen (^1^O_2_), which oxidizes electron‐neutral 4‐(methylthio)aniline residues **114** on the cage to electron‐deficient sulfoxides **115**. This oxidation step is critical because it lowers the stability of **115** within the cage framework, making it susceptible to displacement by 4‐iodoaniline **113**. The oxidized residues are then displaced through subcomponent exchange with 4‐iodoaniline **113**, whose heavy‐atom effect enhances intersystem crossing and thus increases ^1^O_2_ generation. Therefore, this system exhibits characteristic autocatalytic kinetics, where successive incorporation of iodoaniline subcomponents progressively amplifies the rate of photooxidation.

In 2023, Amara and coworkers introduced an example of autocatalysis in photosensitized oxidation (Figure 24, bottom) that was of relevance to organic synthesis.^[^ ^123^ ^]^ Products of oxidized naphthols; naphthoquinones, could accelerate their own formation as they are also triplet sensitizers. Of the five tested dihydroxynaphthalene (DHN) substrates, 1,5‐DHN **116** (82%) and 1,3‐DHN **118** (85%) gave high yields without an external photocatalyst when irradiated at 460 nm. However, 1,6‐ and 1,7‐DHN showed negligible reactivity under 460 nm irradiation compared to 1,3‐DHN **118** and 1,5‐DHN **116**, highlighting the crucial importance of the hydroxyl group position in enabling efficient autocatalysis. The discrepancy between different substitutions could be i) that the product does not sufficiently absorb light at the applied wavelength, or ii) that the starting materials have a lower tendency to oligomerize, and they instead form dark “melanin‐like aggregates”. The authors' kinetic data clearly shows the disappearance of the induction period with increasing amounts of added product, juglone **117**. They evaluated the use of juglone as a singlet oxygen photosensitizer catalyst for the (successful) oxidation of other substrates, such as β‐pinene **120** and furfural **122**.

**Figure 25:**
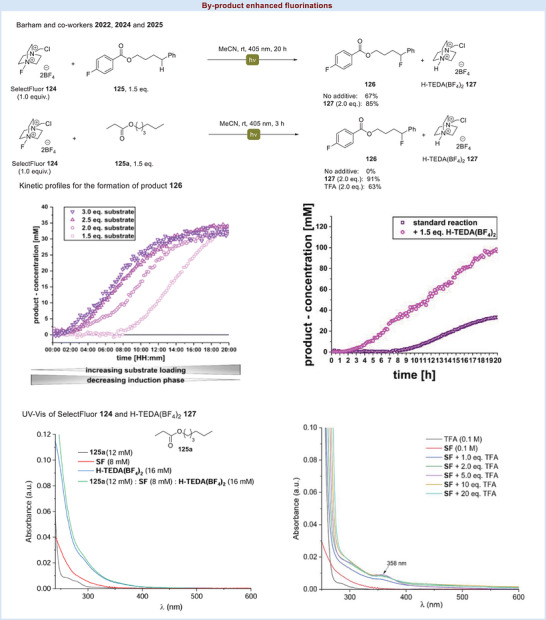
Top, role of the fluorination source by‐product for the fluorinations of unactivated C─H bonds. Middle left, kinetic profile with different 4‐fluorobenzoyl auxiliary‐loaded substrate (**125**) concentrations, reprinted with permission,^[^ ^124^ ^]^ licensed under CC BY 3.0. Copyright © 2022 Royal Society of Chemistry. Middle right, kinetic profile with the addition of H‐TEDA(BF_4_)_2_, is reprinted with permission.^[^ ^125^ ^]^ Copyright © 2024 Wiley‐VCH GmbH. Bottom, UV‐Vis profiles of SelectFluor and additives H‐TEDA(BF_4_)_2_ or TFA are reprinted with permission.^[^ ^126^ ^]^ Copyright © 2025 from the Royal Society of Chemistry.

Photochemical reactions that do not require a photocatalyst can offer milder reaction conditions but may introduce complexity in understanding the mechanism. Over the past few years, our group has investigated the mechanism of direct fluorination of C─H bonds with Selectfluor **124** under photochemical conditions through energy transfer (Figure)^[^ ^124^, ^125^, ^126^ ^]^ Previous studies since >10 years ago (2014 onwards, cited in our studies)^[^ ^121^, ^122^, ^123^ ^]^ had used a multitude of different organic photosensitizers in catalytic loadings to engage Selectfluor in energy transfer. This was proposed to initiate a radical chain mechanism, where in the propagation step, the generated *N*‐(chloromethyl)tetraethylenediamine (TEDA) radical dication engages C─H bonds in HAT, and the resulting C‐centered radicals abstract a fluorine atom from Selectfluor to regenerate the TEDA radical dication. However, such reaction conditions were not successful for any substrates containing free 2° or 1° alcohols or amines. Our group discovered that a 4‐fluorobenzoyl group as a photosensitizing “auxiliary” (as in **125**) allowed to overcome this synthetic hurdle and additionally gave smooth access to reaction kinetics in a robust way with on‐line irradiation within an NMR spectrometer. Surprisingly, an induction period was observed. By increasing the concentration of auxiliary‐loaded substrate, this decreased the induction period (Figure 25, middle left), however did not explain the origin of the induction period to start with.

Subsequent studies revealed that the formation of the by‐product, H‐TEDA(BF_4_)_2_ **127**, played a crucial role in these fluorinations, even without the 4‐fluorobenzoyl auxiliary (kinetics not shown).^[^ ^122^ ^]^ When isolated and added at the start of a “photocatalyst”‐present fluorination reaction, H‐TEDA(BF_4_)_2_ not only cuts down the induction period but also allows reactions to reach higher overall yields of fluorinated products (Figure 25, middle right), suggesting a rate enhancement in the chain propagation step. The UV‐Vis of a mixture of Selectfluor with Brønsted acid‐type additives revealed enhanced charge‐transfer character (TFA was used as a surrogate for H‐TEDA(BF_4_)_2_ to access higher equivalences versus Selectfluor, due to the limiting solubility of H‐TEDA(BF_4_)_2_ in MeCN), tailing into the visible region (Figure 25, bottom).

Ultimately, it was found that by‐product H‐TEDA(BF_4_)_2_ forms a heteroaggregate with Selectfluor, which is photoactive and facilitates C─H fluorination without the need for any exogenous photocatalyst. This revealed that the roles of exogenous “photocatalysts” in previous reports in the field were simply as initiators to generate a small amount of H‐TEDA(BF_4_)_2_ to form the heteroaggregate, which i) takes over as the photoactive species and ii) serves as a more reactive source of fluorine atoms in the propagation step, allowing productive reactivity to outcompete deleterious side reactions as a pathway to higher overall product yields. The discovery could be described as a photo‐autoinductive autocatalytic EDA complex. Overall, our studies of these photochemical C─H fluorination reactions revealed how a sizeable number of previous studies (since 2014 onwards) had overlooked the phenomena of autocatalysis by not investigating reaction kinetics and dismissing the by‐product H‐TEDA(BF_4_)_2_ **127** as an innocent waste product. This case study could represent one of the many examples in the photochemical literature that may involve autocatalysis but not be explicitly described as such.

### 3.2 Photoinduced Electron Transfer: Reductive Quenching

**Figure 26:**
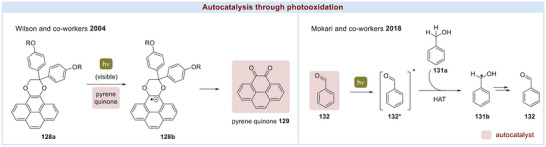
Left, pyrene quinone as an efficient in situ‐generated visible light‐activated oxidant for its own precursor. Right, autocatalytic photooxidation of benzyl alcohol.

In this sub‐section, examples of autocatalysis where an excited‐state photooxidant is quenched reductively are discussed. The term “autocatalytic photooxidation” will be used. Early reports on autocatalytic photooxidation processes included the oxidation of xanthobilirubic acid,^[^ ^127^ ^]^ polyvinyl chloride,^[^ ^128^ ^]^ and hydrogen sulfite oxidation to sulfate.^[^ ^129^ ^]^ Wilson and coworkers reported an example of autocatalysis when a photoexcited product, pyrene quinone **129**, functioned as an electron acceptor to extrude quinone from pyrene dihydrodioxins **128a** (Figure 26, left).^[^ ^130^ ^]^ Recently, autocatalytic photooxidation was observed for the manufacturing of biosurfactants from renewable feedstocks.^[^ ^131^ ^]^

A very common reaction to evaluate the performance and reactivity of (heterogeneous) photocatalysts is the oxidation of benzyl alcohol **131a** to benzaldehyde **132** and the formation of H_2_O_2_. Over the last few years, autocatalytic photooxidation of benzaldehyde has been reported, bringing into question studies where benzyl alcohol was used as an electron donor.^[^ ^132^, ^133^, ^134^ ^]^ One of the reasons for undetected autocatalytic oxidation could arise if the reaction time is shorter than the induction period. Autocatalysis takes place as benzaldehyde **132** is photoexcited to its triplet excited state 132*, (Figure 26, right). This engages in HAT with benzyl alcohol **131a**, resulting in two α‐hydroxybenzyl radicals **131b**, one derived from photoexcited benzaldehyde and the other from benzyl alcohol, which are then intercepted with oxygen. Subsequent steps result in the formation of benzaldehyde (that can re‐enter the photocatalytic cycle) and H_2_O_2_. Such an autocatalytic mechanism was also implicated in the oxidation of diphenylmethanol to produce higher concentrations of H_2_O_2_.^[^ ^135^ ^]^ Extensive mechanistic investigations by Li, Zhu, Mao, and coworkers added another dimension of this benzyl alcohol oxidation.^[^ ^136^ ^]^ Their investigations led to another mechanistic possibility whereby the peroxyl radical acts as a chain carrier. Under these autocatalytic and radical chain mechanisms, they reported oxidation of primary electron‐rich and ‐poor benzyl alcohols to carboxylic acids and secondary electron‐rich and ‐poor benzyl alcohols to ketones.

**Figure 27:**
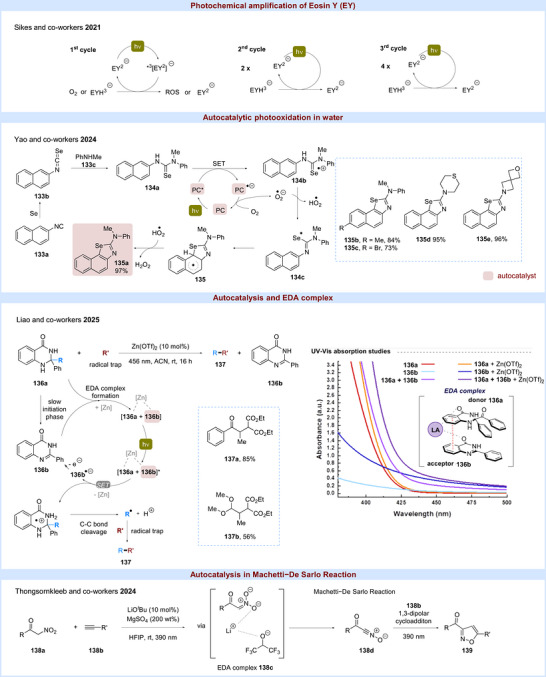
Top, photochemical amplification for the synthesis of Eosin Y. Middle (Yao and coworkers), photooxidation autocatalysis in water for multi‐component tandem reaction for the synthesis of 2‐aminonaphtho[2,1‐d]selenazole derivatives. Middle (Liao and coworkers), repurposing by‐product as an autocatalyst via EDA complex formation, UV‐Vis is reprinted with permission.^[^ ^140^ ^]^ Copyright © 2025 American Chemical Society, CC‐BY 4.0. Bottom, photochemical EDA complex driven Machetti‐De Sario reaction with autocatalysis arising from the by‐product nitrite anion.

In 2021, Sikes and coworkers utilized photoredox autocatalysis to accelerate the formation of a well‐known photocatalyst, Eosin Y, from its non‐fluorescent derivative (EYH^3−^) (Figure 27, top).^[^ ^137^ ^]^ This mode of amplification of Eosin Y allowed it to be used in polymerization and in bioactive assays. Specifically, photoinduced oxidative polymerization of 3,3′‐diaminobenzidine — a fluorescence assay for the detection of SARS‐CoV‐2 nucleocapsid protein — and a colorimetric assay for the detection of streptavidin. The same group also developed a dual photoredox catalysis strategy to enhance the sensitivity of colorimetric bio‐detection in paper‐based diagnostic tests through photopolymerization.^[^ ^138^ ^]^

Recently, Yao and coworkers uncovered photooxidation autocatalysis in multi‐component tandem reactions for the synthesis of 2‐aminonaphtho[2,1‐d]selenazole **135** derivatives in water (Figure 27, middle).^[^ ^139^ ^]^ Intermediate **134a** was formed from Se powder, isonitrile **133a**, and secondary amine **133c**, and participated in SET with a photoexcited species. The photoexcited product was an efficient photooxidant, becoming a catalyst for oxidizing intermediate **134a** to its radical cation **134b**. This radical cation then undergoes further transformations that include SET events with oxygen. The autocatalytic cycle is closed as the reduced form of the PC/product (e.g. radical anion of **135a**) reduces oxygen. The substrate scope demonstrated good to high yields (61–97%) for **135a**‐**135e** with a diverse range of amine partners, including both aliphatic and aromatic amines. The cyanonaphthalene substrates contained both electron‐donating and electron‐withdrawing groups, further speaking to the generality of the chemistry.

Redox auxiliaries are widely used in the field of photochemistry to allow SET events under milder conditions; however, this results in stoichiometric waste. During the peer review of this review manuscript, a report appeared by Liao and coworkers, which uncovered the ‘hidden’ role of a redox auxiliary by‐product in an autocatalytic process (Figure 27, middle).^[^ ^140^ ^]^ Their work demonstrates a photo‐autoinductive autocatalytic EDA (electron‐donor‐acceptor) complex between starting material **136a** and by‐product **136b**. Upon irradiation, pro‐aromatic dihydroquinazolinone (**136a**, DHQZ) undergoes C─C bond cleavage, generating a quinazolinone by‐product **136b**. This by‐product **136b** forms an EDA complex with the starting material DHQZ **136a**, wherein quinazolinone by‐product **136b** is an in situ generated acceptor and DHQZ **136a** is the donor. Formation of this complex is enhanced by the presence of a Lewis acid (Zn(OTf)_2_), which lowers the energy gap and facilitates the SET process. UV‐Vis analysis revealed a bathochromic shift of the complex [**136a **+ **136b**] in comparison to corresponding absorptions of **136a** and **136b** alone. This is shifted even further with the addition of Zn(OTf)_2_.

In a similar vein to how our earlier work repurposed Selectfluor's waste by‐product H‐TEDA(BF_4_)_2_ as a performance additive for the very C─H fluorination reactions that generate it via photo‐autoinductive autocatalysis (Figure 25),^[^ ^123^ ^]^ Liao and coworkers successfully repurposed a redox auxiliary by‐product to enhance the very SET reaction it was generated from.^[^ ^140^ ^]^ This emphasizes the potential of autocatalysis to contribute to green chemistry efforts, particularly its repurposing of waste in a ‘circular chemistry‘ fashion, but also in its exclusion of additional exogenous photocatalysts that prevents further (albeit minor) waste.

Elsewhere, a report by Thongsornkleeb and coworkers described a photochemical Machetti–De Sarlo reaction enabled by an EDA complex for the synthesis of 5‐substituted 3‐acylisoxazoles **139** (Figure 27, bottom).^[^ ^141^ ^]^ Although the reaction exhibits autocatalytic behavior, the autocatalysis is not directly linked to the EDA complex **138c**. Instead, it arises from the decomposition product nitrite anion, which catalyzes the (thermal) downstream conversion of acylnitromethanes to nitrile oxides **138d**. This report is significant in its own right as an example of how thermal autocatalysis can aid a photochemical process (the thermal autocatalysis is sandwiched between two photochemical processes).

### 3.3 Photoinduced Electron Transfer: Oxidative Quenching

**Figure 28:**
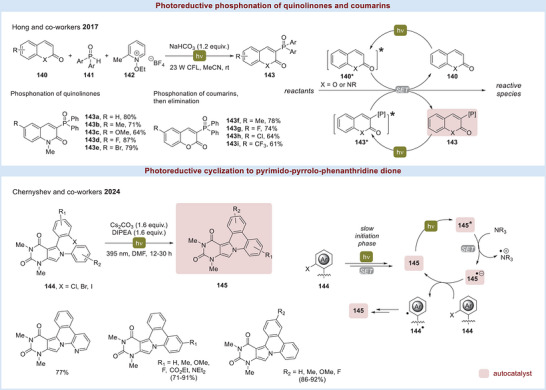
Autocatalysis in photoreductive reactions. Top, phosphonation of quinolinones and coumarins. Bottom, cyclization to pyrimido‐pyrrolo‐phenanthridine dione.

In this sub‐section, examples of autocatalysis where an excited state photoreductant is quenched oxidatively are discussed. Unlike photooxidation, there are only a handful of reports mentioning photoreduction and autocatalysis. Hong and coworkers reported phosphonation of quinolinone and coumarin derivatives wherein both starting material **140** as well as product **143** are efficient photocatalysts to reduce a pyridinium salt **142**. This promoted dissociation of the N─O bond, resulting in an O‐centered radical for HAT needed in the reaction (Figure 28, top).^[^ ^142^ ^]^ Another report on photoreductive autocatalysis was reported by Chernyshev and coworkers, where a highly conjugated system, pyrimido‐pyrrolo‐phenanthridine dione **145**, was formed after C─X bond activation under light irradiation (Figure 28, bottom).^[^ ^143^ ^]^ After the initial formation of a small amount of the product **145**, photoredox autocatalysis took over, as indicated by sigmoidal reaction kinetics and an inhibited induction period upon addition of product at the beginning of the reaction. The authors were able to activate various carbon‐halogen bonds, including chlorine, bromine, and iodine. Photoexcited product 145* is first reduced by an amine to form its radical anion, which then reduces the carbon‐halogen bond of the starting material **144**, resulting in an aryl radical, which then cyclizes to form another molecule of the product (photocatalyst) **145**. As the reaction conditions can activate difficult C─Cl bonds, the authors proposed the possibility of a consecutive PET mechanism.^[^ ^144^, ^145^, ^146^ ^]^ Here, the radical anion of the product is further excited by second photon in order to reach the redox potential required to activate the carbon‐halogen bond. This methodology allowed the formation of pyrimido‐pyrrolo‐phenanthridine diones with various substituents, including electron‐donating and withdrawing groups.

**Figure 29:**
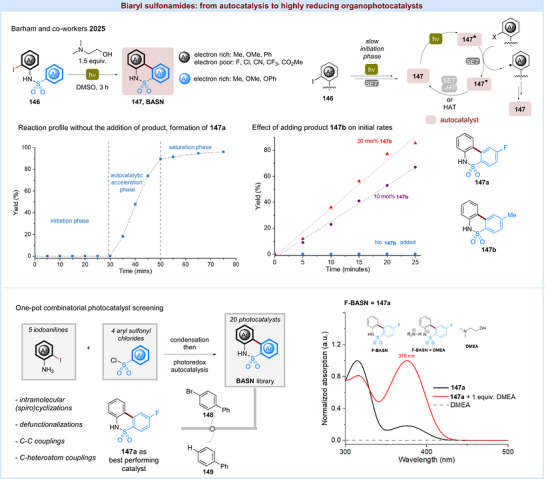
Photoredox autocatalytic formation of biaryl sulfonamides, highly reducing photocatalysts. Kinetic profiles and UV‐Vis are reprinted with permission.^[^ ^119^ ^]^ Copyright © 2025 Wiley‑VCH GmbH. CC BY 4.0

Recently, our group disclosed a definitive example of photoredox autocatalysis for the synthesis of biaryl sulfonamides **147** (BASN) (Figure 29).^[^ ^119^ ^]^ This procedure preferably activated carbon‐iodine bonds in the presence of a labile tosyl group. In the presence of carbon‐bromine bonds, detosylation occurred. Electron‐donating and fluorine groups on the sulfonyl side were tolerated, while electron‐withdrawing groups led to detosylation. The deprotonated product **147** can efficiently reduce the starting material **146** once photoexcited, resulting in an *N*‐centered radical of **147** and an aryl radical. Cyclization of the aryl radical and oxidative aromatization generated another molecule of the product, ready to photocatalyze its own formation. The kinetic profile without the addition of the product at the start of the reaction clearly shows an initiation phase, which disappears once the product is added at the start of the reaction. The UV‐Vis absorption spectra of **147a** (F‐BASN) shows that it absorbs in the near‐visible region and deprotonation results in an enhancement of that absorption, tailing toward 450 nm.

Investigation of the photophysical and electronic properties of BASN showed that it is a highly reducing photocatalyst with excited state redox potentials of approximately −2.31 V (**147a**) and −2.45 V (**147b**) versus SCE. We then conducted one‐pot combinatorial synthesis of a BASN library directly from commercial iodoanilines and sulfonyl chlorides under photoredox autocatalytic conditions, resulting in 20 different photocatalysts with varied electronics. These in situ‐generated photocatalysts were able to reduce 4‐bromobiphenyl **148** (−2.4 V vs. SCE). This approach resulted in **147a** being identified as the best‐performing photocatalyst, which reductively dehalogenated 4‐bromobiphenyl **148**, affording a 74% yield of biphenyl **149**. Afterwards, the photocatalytic potential of BASN was showcased in many more photoreductive transformations, such as intramolecular (spiro)‐cyclizations, defunctionalizations, and C─C / C─heteroatom couplings.

### 3.4 Transition Metal‐Constrained Auto‐Photocatalysis

**Figure 30:**
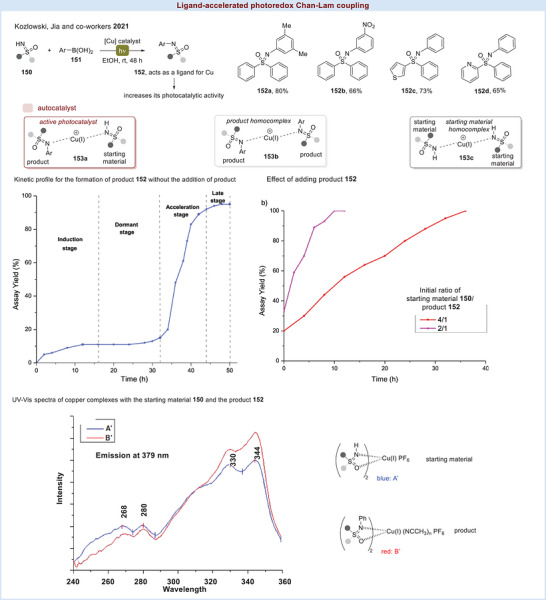
Product acts as a ligand to improve photochemical properties of Cu‐catalyst. Kinetic and UV‐vis profiles are reprinted with permission.^[^ ^147^ ^]^ Copyright © 2021, Springer Nature, CC BY 4.0.

Kozloswki, Jia, and coworkers discovered an auto‐photocatalytic Chan‐Lam coupling for *N*‐arylation of NH‐sulfoximines **150** with arylboronic acids **151** using a copper catalyst. The product **152** acted as a ligand for the copper species, rendering it a photocatalyst for C─N couplings (Figure 30).^[^ ^147^ ^]^ Though the diaryl sulfoximine starting material **150** could also serve as a ligand, kinetic studies revealed that the heterocomplex **153a** — with both starting material and product ligated to copper — accelerated the reaction more effectively as a photocatalyst compared to either product **153b** or starting material homocomplex **153c**. Moreover, the kinetic profile revealed an induction period, which is absent upon addition of product at the start of the reaction. Characteristics of the UV‐Vis absorption spectra of copper complexes with the starting material and the product were very similar. This cannot therefore be considered a “pure” autocatalysis, since the maximum catalyst loading will ultimately be constrained by the initial loading of copper salt (10 mol%), beyond which the reaction must switch from an autophotocatalytic to a photocatalytic regime. Potentially, as the concentration of product increases, it forces the speciation of copper complexes toward the homocomplex with two product molecules as ligands, which is a less effective catalyst. Overall, we refer to this mechanistic paradigm as “transition metal‐constrained” auto‐photocatalysis.

In terms of the mechanism, the authors proposed that upon the excited state coordinating EtOH solvent and undergoing MLCT, H_2_ evolution occurs, which was detected in the headspace of the reaction vessel. Transmetalation of the boronic acid and reductive elimination afford the product, that can go on to form another heterocomplex. The reaction can be considered net‐oxidative overall. Although no stoichiometric oxidant was required, H_2_ evolution occurred involving EtOH as an H atom donor. A broad range of sulfoximines and boronic acids were successfully coupled, suggesting the auto‐photocatalysis was robust enough to withstand electronic alterations to the starting partners.

The authors discovered the photoactivity of the heterocomplex by excluding ambient light from the reaction, however no deliberate irradiation with a lamp or monochromatic LED was done to confirm the wavelength needed for reactivity. This may explain the long induction periods and employed reaction times of 32 (Figure 30, middle) and 48 hours (Figure 30, top), respectively. The UV‐Vis absorption spectra for copper complexes of starting material and product show that their maximum absorption wavelengths are similar (Figure 30, bottom). Photophysical characterization of the copper heterocomplex was limited; likely complicated by the speciation of the complex with its respective homocomplexes involving either two molecules of starting material or two molecules of product as ligands. The copper heterocomplexes were not explored as exogenous photocatalysts in other transformations, to evaluate if they could be used, e.g., for outer‐sphere electron transfer reactions or in copper photocatalyses.^[^ ^148^, ^149^, ^150^ ^]^ While the aforementioned aspects require attention in order to confirm a broader utility, this transition metal‐constrained example offers a highly strategic design principle for auto‐photocatalysis. With many poly‐ligated transition metal complexes being photoactive (e.g. prototypical Ru or Ir polypyridyl complexes), one could envisage planned autophotoredox catalyses to predictably synthesize target products as ligands, that could be post‐de‐ligated. However, this may require metal salt precursors/complexes employed stoichiometrically.

### 3.5 Radicals in Biological Systems

**Figure 31:**
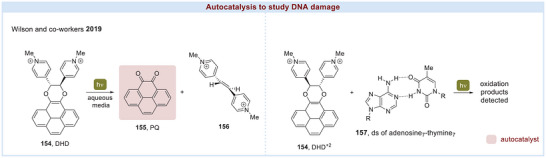
On the examination of DNA damage sequence coupled with photo(auto)catalysis.

Biological systems were also demonstrated to follow photochemically mediated autocatalytic processes (not shown). For example, Mokhir and coworkers demonstrated autocatalytic oxidation of 2′,7′‐dichlorofluorescein was dramatically enhanced by an added nucleic acid photosensitizer.^[^ ^151^ ^]^ Additionally, a singlet oxygen‐mediated autocatalysis process was confirmed in the photorelease of a sensitizer drug bound to a fluorinated silica support. In that system, irradiation of the released sensitizer accelerated the formation of singlet oxygen, which, in‐turn, photoreleased more sensitizer.^[^ ^152^ ^]^ Autocatalytic photooxidation was utilized to enhance the sensitivity of chemifluorescence‐based enzyme‑linked immunosorbent assay. The chemifluorescent substrate, 10‐acetyl 3,7‐dihydroxyphenoxazine, photooxidized to give a fluorescent product resorufin.^[^ ^153^ ^]^ Skorb and coworkers combined enzymatic autocatalysis with photocatalysis to regulate an autocatalytic wave of enzymatic reactions in a hydrogel medium. Photocatalytic oxidation/reduction on TiO_2_ induced acidification under irradiation, which suppressed the autocatalytic conversion of trypsinogen to trypsin, demonstrating light‐controlled regulation of enzymatic networks.^[^ ^154^ ^]^ Wilson and coworkers investigated DNA oxidation using dipyridinium dihydrodioxin **154** (DHD), to make pyrene quinone **155** (PQ) water soluble (Figure 31). Photochemical release of pyrene quinone **155** from nucleic acids, though still intercalated in the DNA base pair stack, can further oxidize (damage) DNA under light irradiation. This technique can be used to precisely examine how the sequence was damaged (Figure 31).^[^ ^155^ ^]^

Otto and co‐workers designed a photocatalytic synthetic self‐replicating system using dyes as co‐factors to transform thiols into disulfide precursors. These disulfide precursors are then responsible for the growth of replicators under light irradiation.^[^ ^156^ ^]^ Gong and co‐workers addressed an inherent problem in reactive oxygen species(ROS)‐based cancer treatment; the insufficient presence of oxy‐substrates in tumor microenvironments. Therefore, the authors designed an autocatalytic nanoreactor was designed which self‐supplied hydrogen peroxide and oxygen to improve ROS‐based chemo/photodynamic therapy.^[^ ^157^ ^]^ In general, autocatalysis in biological systems is well‐studied, and these examples also show the key influence of auto(photo)catalytic processes in biological systems.

### 3.6 Non‐Photochemical Open‐Shell Autocatalytic Reactions

**Figure 32:**
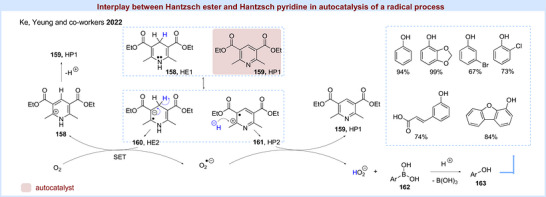
Hantzsch ester and Hantzsch pyridine in an autocatalytic cycle.

Non‐photochemical open‐shell autocatalyses are rare. One of the examples came from the group of Ke, Yeung, and co‐workers, who explored the ability of Hantzsch esters (HE) as transfer hydrogenation agents, engaging pyridine to generate a reactive radical ion intermediate pair that ultimately leads to hydroxylation of arylboronic acids (Figure 32). Though not originally proposed by the authors, we expect exchange of a hydride between this pair would afford^[^ ^158^ ^]^ radical **160** (HE2) and radical **161** (HP2) (Figure 32). The authors leveraged aerobic conditions to allow SET from radical **160** to O_2_ to form superoxide anion and pyridine **159** (upon deprotonation). These reacted with radical **161** to form hydroperoxide anion and pyridine **159**. Nucleophilic addition of hydroperoxide anion to the boron atom of the arylboronic acid, followed by 1,2‐boron shift and hydrolysis affords *ipso*‐hydroxylated product **163**. Although **158** and **159** are not the actual final products, their interplay is autocatalytic. This methodology allowed the use of exceptionally mild and aerobic reaction conditions without employing metal‐based catalysts for the formation of both electron‐rich and electron‐poor phenols.

Liu and Chen later identified another distinct example of open‐shell autocatalysis during the early pyrolysis of 1,3,5‐trinitro‐1,3,5‐triazine (RDX).^[^ ^159^ ^]^ Their computational study revealed that the NO_2_^−^ ion facilitates denitration through proton‐coupled electron transfer whereby protons from the methylene groups of RDX and electrons from the *N*‐heterocycle are transferred to the NO_2_^−^ ion, forming HONO and another NO_2_^−^ ion. This regeneration of the NO_2_^−^ ion enables an autocatalytic denitration pathway that is both kinetically and thermodynamically favorable under high‐temperature, non‐photochemical conditions.

## 4 Summary and Outlook

The literature on autocatalysis is vast and very interdisciplinary, revealing surprising insights into both well‐established systems and new reactivities. From biochemical systems to synthetic catalysis, autocatalytic mechanisms play a crucial role in driving efficiency and selectivity. Most of the literature on autocatalysis is a result of thorough mechanistic investigations. A rational design approach would instead allow to plan new autocatalytic reactions. For example, ligand‐accelerated catalysis could be used to enhance reaction efficiency and unlock novel transformations. In terms of photocatalysis, a product could be designed with extended conjugation to absorb visible light and actively participate in redox reactions. Moreover, integrating autocatalytic systems into continuous‐flow reactors and exploring their initiation via electrochemical systems could improve scalability and sustainability by promoting self‐accelerating reaction pathways under mild reaction conditions.

## Conflict of Interest

The authors declare no conflict of interest.

## Data Availability

Data sharing is not applicable to this article as no new data were created or analyzed in this study.

## References

[1] T. A. Lincoln , G. F. Joyce , Science 2009, 323, 1229. 19131595 10.1126/science.1167856 PMC2652413

[2] M. G. Howlett , S. P. Fletcher , Nat. Rev. Chem. 2023, 7, 673. 37612460 10.1038/s41570-023-00524-8

[3] R. S. Jack , F. Scholz , Wilhelm Ostwald, Springer International Publishing, Cham, 2017.

[4] W. Ostwald , “Über Autokatalyse”, can be found under https://archiv.saw‐leipzig.de/publikationen‐quellen/publikationen/berichte‐ueber‐die‐verhandlungen‐der‐koeniglich‐saechsischen‐gesellschaft‐der‐wissenschaften‐zu‐leipzig‐mathematisch‐physische‐klasse‐bd‐1/berichte‐ueber‐die‐verhandlungen‐der‐koeniglich‐saechsischen‐gesellschaft‐der‐wissenschaften‐zu‐leipzig‐mathematisch‐physische‐klasse‐bd‐42/w‐ostwald‐ueber‐autokatalyse, 2022.

[5] NobelPrize.org, “Nobel Prize in Chemistry 1909”, can be found under https://www.nobelprize.org/prizes/chemistry/1909/summary/, 2025.

[6] W. Hordijk , J. Theor. Biol. 2017, 435, 22. 28888946 10.1016/j.jtbi.2017.09.003

[7] C. de Rocafiguera , V. Lloveras , J. Vidal‐Gancedo , J. Teixidó , R. Estrada‐Tejedor , J. I. Borrell , R. La Puig de Bellacasa , Org. Chem. Front. 2023, 11, 27.

[8] P. Yadav , D. Chatterjee , S. Bhowmick , K. Tiwari , A. Awasthi , D. K. Tiwari , Chem. Commun. 2024, 60, 12746. 10.1039/d4cc04044a 39397744

[9] I. K. Goncharova , S. A. Filatov , A. P. Drozdov , A. A. Tereshchenko , P. A. Knyazev , A. A. Guda , I. P. Beletskaya , A. V. Arzumanyan , J. Catal. 2024, 429, 115269.

[10] L. Tang , F. Jia , R. Yu , L. Zhang , Q. Zhou , Org. Biomol. Chem. 2024, 23, 151. 39513995 10.1039/d4ob01585a

[11] S. Liu , H. Liao , B. Chen , T. Guo , Z. Zhang , H. Lin , Green Chem. 2024, 26, 10456.

[12] A. J. Ward , C. C. Weber , A. F. Masters , T. Maschmeyer , ChemCatChem 2013, 5, 959.

[13] J. K. G. Karlsson , O. J. Woodford , R. Al‐Aqar , A. Harriman , J. Phys. Chem. A 2017, 121, 8569. 29048161 10.1021/acs.jpca.7b06440

[14] Y. Wu , R. Chen , H. Liu , Y. Wei , D. Wu , J. Nanosci. Nanotechnol. 2014, 14, 7325. 25924410 10.1166/jnn.2014.8979

[15] Z. Cao , T. Zhao , X. Yang , W. Jiang , K. Nie , W. Xia , X. Wang , L. Wang , C. Zhou , Y. Zhang , G. Han , H. Ben , Ind. Crop. Prod. 2024, 209, 118068.

[16] Y. Battie , N. Destouches , L. Bois , F. Chassagneux , A. Tishchenko , S. Parola , A. Boukenter , J. Phys. Chem. C 2010, 114, 8679.

[17] K. Akamatsu , A. Kimura , H. Matsubara , S. Ikeda , H. Nawafune , Langmuir 2005, 21, 8099. 16114908 10.1021/la051298f

[18] R. M. Garcia , Y. Song , R. M. Dorin , H. Wang , P. Li , Y. Qiu , F. van Swol , J. A. Shelnutt , Chem. Commun. 2008, 2535. 10.1039/b801695j 18506235

[19] L. C. Mayer , S. Heitsch , O. Trapp , Acc. Chem. Res. 2022, 55, 3345. 36351215 10.1021/acs.accounts.2c00557

[20] A. I. Hanopolskyi , V. A. Smaliak , A. I. Novichkov , S. N. Semenov , ChemSystemsChem 2021, 3, e2000026.

[21] J. F. Scholtes , O. Trapp , Synlett 2021, 32, 971.

[22] P. Schuster , Monatsh. Chem. 2019, 150, 763.

[23] G. Clixby , L. Twyman , Org. Biomol. Chem. 2016, 14, 4170. 27086507 10.1039/c6ob00280c

[24] A. J. Bissette , S. P. Fletcher , Angew. Chem. Int. Ed. 2013, 52, 12800. 10.1002/anie.201303822 24127341

[25] A. Vidonne , D. Philp , Eur. J. Org. Chem. 2009, 2009, 593.

[26] D. G. Blackmond , Angew. Chem. Int. Ed. 2009, 48, 386. 10.1002/anie.200804565 19053125

[27] A. K. Horváth , Phys. Chem. Chem. Phys. 2021, 23, 7178. 33734272 10.1039/d1cp00224d

[28] R. Plasson , A. Brandenburg , L. Jullien , H. Bersini , J. Phys. Chem. A 2011, 115, 8073. 21650179 10.1021/jp110079p

[29] A. Butlerow , Justus Liebigs Ann. Chem. 1861, 120, 295.

[30] R. Breslow , Tetrahedron Lett. 1959, 1, 22.

[31] D. R. Nadkarni , S. M. Mehta , T. S. Wheeler , J. Phys. Chem. 1935, 39, 727.

[32] M. J. White , F. J. Leeper , J. Org. Chem. 2001, 66, 5124. 11463265 10.1021/jo010244h

[33] R. S. Massey , J. Murray , C. J. Collett , J. Zhu , A. D. Smith , A. C. O'Donoghue , Org. Biomol. Chem. 2021, 19, 387. 33351015 10.1039/d0ob02207a

[34] R. J. Field , E. Koros , R. M. Noyes , J. Am. Chem. Soc. 1972, 94, 8649.

[35] L. Howell , E. Osborne , A. Franklin , É. Hébrard , J. Phys. Chem. B 2021, 125, 1667. 33534567 10.1021/acs.jpcb.0c11079 PMC7898267

[36] J. L. Hudson , J. C. Mankin , J. Chem. Phys. 1981, 74, 6171.

[37] A. M. Zhabotinsky , Chaos 1991, 1, 379. 12779934 10.1063/1.165848

[38] J. A. Pojman , D. C. Leard , W. West , J. Am. Chem. Soc. 1992, 114, 8298.

[39] R. P. Washington , W. W. West , G. P. Misra , J. A. Pojman , J. Am. Chem. Soc. 1999, 121, 7373.

[40] M. ter Harmsel , O. R. Maguire , S. A. Runikhina , A. S. Y. Wong , W. T. S. Huck , S. R. Harutyunyan , Nature 2023, 621, 87. 37673989 10.1038/s41586-023-06310-2 PMC10482680

[41] T. Amemiya , J. Wang , J. Phys. Chem. A 2010, 114, 13347. 21138287 10.1021/jp108186q

[42] R. O. Pires , R. B. Faria , Inorg. Chem. 2022, 61, 1178. 34965359 10.1021/acs.inorgchem.1c03522

[43] D. Ratna , Handbook of Thermoset Resins, iSmithers, Shawbury, Shrewsbury, Shropshire, 2009.

[44] J.‐Y. Lee , M.‐J. Shim , S.‐W. Kim , Mater. Chem. Phys. 1997, 48, 36.

[45] Y. Liu , Z. Yu , B. Wang , P. Li , J. Zhu , S. Ma , Green Chem. 2022, 24, 5691.

[46] M. A. Bashir , Coatings 2023, 13, 592.

[47] F. Muench , ChemElectroChem 2021, 8, 2993.

[48] R. Muraliraja , R. Anthoni Sagaya Selvan , A. Selvakumar , M. Franco , T. R. Tamilarasan , U. Sanjith , W. Sha , J. Sudagar , J. Alloys Compd. 2023, 960, 170723.

[49] N. V. Muravyev , M. V. Gorn , I. N. Melnikov , K. A. Monogarov , B. L. Korsunskii , I. L. Dalinger , A. N. Pivkina , V. G. Kiselev , Phys. Chem. Chem. Phys. 2022, 24, 16325. 35758846 10.1039/d1cp04663b

[50] N. V. Muravyev , D. K. Pronkin , M. S. Klenov , A. A. Voronin , I. L. Dalinger , K. A. Monogarov , Phys. Chem. Chem. Phys. 2023, 25, 3666. 36648387 10.1039/d2cp05759j

[51] K. Wang , D. Liu , S. Xu , G. Cai , J. Loss Prev. Process Ind. 2016, 40, 241.

[52] C. Wei , W. J. Rogers , M. S. Mannan , J. Therm. Anal. Calorim. 2006, 83, 125.

[53] F. C. Frank , Biochim. Biophys. Acta 1953, 11, 459. 13105666 10.1016/0006-3002(53)90082-1

[54] D. G. Blackmond , C. R. McMillan , S. Ramdeehul , A. Schorm , J. M. Brown , J. Am. Chem. Soc. 2001, 123, 10103. 11592892 10.1021/ja0165133

[55] K. Soai , T. Shibata , H. Morioka , K. Choji , Nature 1995, 378, 767.

[56] K. Soai , S. Niwa , H. Hori , J. Chem. Soc., Chem. Commun. 1990, 982.

[57] K. Soai , T. Hayase , K. Takai , Tetrahedron: Asymmetry 1995, 6, 637.

[58] K. Soai , T. Kawasaki , A. Matsumoto , Asymmetric Autocatalysis: The Soai Reaction, The Royal Society of Chemistry, 2022.

[59] D. G. Blackmond , Chem. Rev. 2020, 120, 4831. 31797671 10.1021/acs.chemrev.9b00557

[60] T. Shibata , S. Yonekubo , K. Soai , Angew. Chem. Int. Ed. 1999, 38, 659. 10.1002/(SICI)1521-3773(19990301)38:5<659::AID-ANIE659>3.0.CO;2-P 29711547

[61] M. Quaranta , T. Gehring , B. Odell , J. M. Brown , D. G. Blackmond , J. Am. Chem. Soc. 2010, 132, 15104. 20942400 10.1021/ja103204w

[62] A. Matsumoto , T. Abe , A. Hara , T. Tobita , T. Sasagawa , T. Kawasaki , K. Soai , Angew. Chem. Int. Ed. 2015, 54, 15218. 10.1002/anie.201508036 PMC4691336 26494200

[63] J. Klankermayer , I. D. Gridnev , J. M. Brown , Chem. Commun. 2007, 3151. 10.1039/b705978g 17653371

[64] I. D. Gridnev , A. K. Vorobiev , ACS Catal. 2012, 2, 2137.

[65] I. D. Gridnev , J. M. Serafimov , H. Quiney , J. M. Brown , Org. Biomol. Chem. 2003, 1, 3811. 14649913 10.1039/b307382n

[66] F. G. Buono , D. G. Blackmond , J. Am. Chem. Soc. 2003, 125, 8978. 15369330 10.1021/ja034705n

[67] S. V. Athavale , A. Simon , K. N. Houk , S. E. Denmark , Nat. Chem. 2020, 12, 412. 32203445 10.1038/s41557-020-0421-8 PMC7117993

[68] S. V. Athavale , A. Simon , K. N. Houk , S. E. Denmark , J. Am. Chem. Soc. 2020, 142, 18387. 33108874 10.1021/jacs.0c05994 PMC7597373

[69] O. Trapp , S. Lamour , F. Maier , A. F. Siegle , K. Zawatzky , B. F. Straub , Chem. Eur. J. 2020, 26, 15871. 32822103 10.1002/chem.202003260 PMC7756584

[70] P. Möhler , G. Betzenbichler , L. Huber , A. Siegle , O. Trapp , Nat Commun. 2025, 16, 7303. 40775212 10.1038/s41467-025-62591-3 PMC12332059

[71] N. Chinkov , A. Warm , E. M. Carreira , Angew. Chem. Int. Ed. 2011, 50, 2957. 10.1002/anie.201006689 21365723

[72] E. S. Saigitbatalova , L. Z. Latypova , A. A. Zagidullin , A. R. Kurbangalieva , I. D. Gridnev , Int. J. Mol. Sci. 2023, 24, 17048. 38069371 10.3390/ijms242317048 PMC10707151

[73] M. Funes‐Maldonado , B. Sieng , M. Amedjkouh , Org. Lett. 2016, 18, 2536. 27176923 10.1021/acs.orglett.6b00742

[74] G. Storch , O. Trapp , Nat. Chem. 2017, 9, 179. 28282051 10.1038/nchem.2638

[75] M. T. Zambri , T. R. Hou , S. Jdanova , M. S. Taylor , ACS Catal. 2024, 14, 6728.

[76] S. N. Semenov , L. Belding , B. J. Cafferty , M. P. S. Mousavi , A. M. Finogenova , R. S. Cruz , E. V. Skorb , G. M. Whitesides , J. Am. Chem. Soc. 2018, 140, 10221. 30035540 10.1021/jacs.8b05048

[77] K. J. Singh , A. C. Hoepker , D. B. Collum , J. Am. Chem. Soc. 2008, 130, 18008. 19053473 10.1021/ja807331k PMC2643095

[78] A. C. Hoepker , L. Gupta , Y. Ma , M. F. Faggin , D. B. Collum , J. Am. Chem. Soc. 2011, 133, 7135. 21500823 10.1021/ja200906z PMC3102585

[79] L. Gupta , A. C. Hoepker , Y. Ma , M. S. Viciu , M. F. Faggin , D. B. Collum , J. Org. Chem. 2013, 78, 4214. 23270408 10.1021/jo302408r PMC3644364

[80] J. Liang , A. C. Hoepker , A. M. Bruneau , Y. Ma , L. Gupta , D. B. Collum , J. Org. Chem. 2014, 79, 11885. 25000303 10.1021/jo501392r PMC4275155

[81] J. Liang , A. C. Hoepker , R. F. Algera , Y. Ma , D. B. Collum , J. Am. Chem. Soc. 2015, 137, 6292. 25900574 10.1021/jacs.5b01668 PMC4788392

[82] J. Cha , E. Lee , D. V. Yandulov , Inorg. Chem. 2022, 61, 14544. 36050901 10.1021/acs.inorgchem.2c01139

[83] J. F. C. Perez , F. L. Kirlin , E. F. Reynolds , C. E. Altomare‐Jarczyk , B. T. Joseph , J. M. Keith , A. R. Chianese , ACS Catal. 2024, 14, 16497. 39507487 10.1021/acscatal.4c04475 PMC11536344

[84] A. Paparo , T. Schindler , J. van Leusen , J. Cook , T. P. Spaniol , P. Kögerler , M. Temprado , C. C. Cummins , J. Okuda , Organometallics 2025, 44, 529.

[85] H. Fujisawa , T. Nakagawa , T. Mukaiyama , Adv. Synth. Catal. 2004, 346, 1241.

[86] W. Cullen , A. J. Metherell , A. B. Wragg , C. G. P. Taylor , N. H. Williams , M. D. Ward , J. Am. Chem. Soc. 2018, 140, 2821. 29412665 10.1021/jacs.7b11334

[87] M. Mauksch , S. B. Tsogoeva , I. M. Martynova , S. Wei , Angew. Chem. Int. Ed. 2007, 46, 393. 10.1002/anie.200603517 17146824

[88] M. Amedjkouh , M. Brandberg , Chem. Commun. 2008, 3043. 10.1039/b804142n 18688342

[89] X. Wang , Y. Zhang , H. Tan , Y. Wang , P. Han , D. Z. Wang , J. Org. Chem. 2010, 75, 2403. 20196532 10.1021/jo902500b

[90] A. Wessels , M. Klussmann , M. Breugst , N. E. Schlörer , A. Berkessel , Angew. Chem. Int. Ed. 2022, 61, e202117682. 10.1002/anie.202117682 PMC9325009 35238462

[91] V. Klein , F. Schuster , J. Amthor , H. Maid , P. Bijalwan , F. Himo , S. Santoro , S. B. Tsogoeva , Angew. Chem. Int. Ed. 2025, e202505275. 10.1002/anie.202505275 40271817

[92] V. K. Aggarwal , S. Y. Fulford , G. C. Lloyd‐Jones , Angew. Chem. Int. Ed. 2005, 44, 1706. 10.1002/anie.200462462 15693043

[93] M.‐L. Tan , M. Ángeles Gutiérrez López , N. Sakai , S. Matile , Angew. Chem. Int. Ed. 2023, 62, e202310393. 10.1002/anie.202310393 37574867

[94] J. K. S. Hansen , C. G. Tortzen , P. G. Sørensen , M. B. Nielsen , Chem. Eur. J. 2023, 29, e202202833. 36217899 10.1002/chem.202202833 PMC10099493

[95] L. Lemiègre , Y. Trolez , Asian J. Org. Chem. 2023, 12, e202300321.

[96] B. J. Paul‐Gorsline , Org. Process Res. Dev. 2024, 28, 2481.

[97] N. March , B. J. Paul‐Gorsline , Org. Process Res. Dev. 2024, 28, 2488.

[98] Q. Duez , L. Marek , J. Váňa , J. Hanusek , J. Roithová , Chem. Eur. J. 2024, 30, e202303619. 38088237 10.1002/chem.202303619

[99] L. Schrecker , J. Dickhaut , C. Holtze , P. Staehle , M. Vranceanu , K. Hellgardt , K. K. Hii , React. Chem. Eng. 2022, 8, 41.

[100] G. Gastelu , P. Saha , P. J. Dyson , M. Hulla , J. G. Uranga , ChemCatChem 2023, 15,e202300905.

[101] K. Ichimura , Chem Rec. 2002, 2, 46. 11933261 10.1002/tcr.10013

[102] R. Kürsteiner , G. Panzarasa , ChemSystemsChem 2023, 5, e202300020.

[103] W. Bai , D. Zhang , X. Wen , Org. Process Res. Dev. 2022, 26, 3042.

[104] K. A. Miller , E. G. Morado , S. R. Samanta , B. A. Walker , A. Z. Nelson , S. Sen , D. T. Tran , D. J. Whitaker , R. H. Ewoldt , P. V. Braun , S. C. Zimmerman , J. Am. Chem. Soc. 2019, 141, 2838. 30698426 10.1021/jacs.8b07705

[105] J. Fu , L. Yi , K. Li , Y. Zhang , T. Zhang , A. Li , T. Wang , Y. Zou , X. Li , H. Yao , Chem. Eng. J. 2025, 506, 159906.

[106] N. S. Sapienza , K. N. Knight , M. Albrahim , M. R. Yousuf , A. M. Karim , J. R. Morris , ACS Catal. 2023, 13, 9997.

[107] K. Li , L. Li , X. Chang , X. Shi , X. Li , C. Pei , Z.‐J. Zhao , J. Gong , Chem Bio Eng 2024, 1, 274. 10.1021/cbe.3c00124 PMC11835173 39974201

[108] R. E. Coleman , R. B. Boulton , A. A. Stuchebrukhov , J. Phys. Chem. B 2023, 127, 4300. 37162385 10.1021/acs.jpcb.3c02172 PMC10201527

[109] P. Hui , M. Branca , B. Limoges , F. Mavré , Chem. Commun. 2021, 57, 11374. 10.1039/d1cc05121k 34647564

[110] Z. Szalai , P. Ábrányi‐Balogh , G. Keglevich , J. Org. Chem. 2025, 90, 439. 39686731 10.1021/acs.joc.4c02355 PMC11731303

[111] D. Lu , K. Wu , T.‐B. Wen , W. Hao , H.‐J. Zhang , J. Org. Chem. 2023, 88, 17494. 37987772 10.1021/acs.joc.3c01300

[112] J. B. Curley , C. Hert , W. H. Bernskoetter , N. Hazari , B. Q. Mercado , Inorg. Chem. 2022, 61, 643. 34955015 10.1021/acs.inorgchem.1c03372

[113] V. Rautenstrauch , P. Megard , B. Bourdin , A. Furrer , J. Am. Chem. Soc. 1992, 114, 1418.

[114] C. Chapuis , B. Winter , K. H. Schulte‐Elte , Tetrahedron Lett. 1992, 33, 6135.

[115] H. B. Kagan , Recl. Trav. Chim. Pays‐Bas 1995, 114, 203.

[116] P. L. H. Mok , B. P. Roberts , P. T. McKetty , J. Chem. Soc., Perkin Trans. 2 1993, 665.

[117] V. Schurig , in Differentiation of Enantiomers I. Topics in Current Chemistry, Vol. 340 (Ed: V. Schurig ), Springer, Cham 2013.

[118] G. Ciamician , Science 1912, 36, 385. 17836492 10.1126/science.36.926.385

[119] J. Kaur , M. J. P. Mandigma , N. A. Bapat , J. P. Barham , Angew. Chem. Int. Ed. 2025, e202423190. 10.1002/anie.202423190 39963861

[120] K. K. Rohatgi‐Mukherjee , A. K. Gupta , Indian J. Chem. 1976, 14A, 723.

[121] J. Wang , H. Li , Y. Cai , D. Wang , L. Bian , F. Dong , H. Yu , Y. He , Anal. Chem. 2019, 91, 6155. 30990015 10.1021/acs.analchem.9b00759

[122] P. P. Neelakandan , A. Jiménez , J. D. Thoburn , J. R. Nitschke , Angew. Chem. Int. Ed. 2015, 54, 14378. 10.1002/anie.201507045 26437971

[123] M. Lancel , P. Zimberlin , C. Gomez , M. Port , L. Khrouz , C. Monnereau , Z. Amara , J. Org. Chem. 2023, 88, 6498. 36988615 10.1021/acs.joc.2c03014

[124] S. Yakubov , W. J. Stockerl , X. Tian , A. Shahin , M. J. P. Mandigma , R. M. Gschwind , J. P. Barham , Chem. Sci. 2022, 13, 14041. 36540818 10.1039/d2sc05735b PMC9728569

[125] S. Yakubov , B. Dauth , W. J. Stockerl , W. da Silva , R. M. Gschwind , J. P. Barham , ChemSusChem 2024, 17, e202401057. 38874542 10.1002/cssc.202401057 PMC11632574

[126] S. Yakubov , J. P. Barham , Org. Chem. Front. 2025, 12, 3156.

[127] J. O. Grunewald , J. C. Walker , E. R. Strope , Photochem. Photobiol. 1976, 24, 29. 972935 10.1111/j.1751-1097.1976.tb06794.x

[128] J. Verdu , J. Macromol. Sci. Part A Chem. 1978, 12, 551.

[129] A. Ansari , J. Peral , X. Domènech , R. Rodríguez‐Clemente , J. Casado , J. Mol. Catal. A Chem. 1996, 112, 269.

[130] E. T. Mack , A. B. Carle , J. T.‐M. Liang , W. Coyle , R. M. Wilson , J. Am. Chem. Soc. 2004, 126, 15324. 15563127 10.1021/ja0473788

[131] E. Padoan , F. Contillo , M. Marafante , E. Montoneri , M. Francavilla , S. Berto , A. Baglieri , Polymers 2024, 16, 1479. 38891426 10.3390/polym16111479 PMC11174893

[132] M. J. Pavan , H. Fridman , G. Segalovich , A. I. Shames , N. G. Lemcoff , T. Mokari , ChemCatChem 2018, 10, 2541.

[133] I. Krivtsov , A. Vazirani , D. Mitoraj , R. Beranek , ChemCatChem 2023, 15, e202201215.

[134] B. C. Moon , B. Bayarkhuu , K. A. I. Zhang , D. K. Lee , J. Byun , Energy Environ. Sci. 2022, 15, 5082.

[135] Q. Miao , M. Liu , Y. Mou , Y. Zhang , Q. Li , Z. Cao , W. Jiang , ACS Sustain. Chem. Eng. 2024, 12, 5596.

[136] X.‐Y. Wang , H.‐E. Lao , H.‐Y. Zhang , Y. Wang , Q. Zhang , J.‐Q. Wu , Y.‐F. Li , H.‐J. Zhu , J.‐Y. Mao , Y. Pan , Molecules 2024, 29, 3429. 39065007 10.3390/molecules29143429 PMC11279666

[137] S. Kim , A. Martínez Dibildox , A. Aguirre‐Soto , H. D. Sikes , J. Am. Chem. Soc. 2021, 143, 11544. 34288684 10.1021/jacs.1c04236

[138] S. Kim , H. D. Sikes , ACS Appl. Mater. Interfaces 2021, 13, 57962. 34797978 10.1021/acsami.1c17589

[139] X. Li , H. Yao , Chin. J. Org. Chem. 2024, 44, 660.

[140] C.‐L. Chan , Y.‐T. Tsao , A. S. Paculba , P.‐S. Lin , Z.‐N. Tsai , H.‐H. Chiu , R. Kunitake , M.‐J. Chiu , C.‐C. Yeh , C.‐C. Chiu , H.‐H. Liao , Org. Lett. 2025, 27, 9593. 40854869 10.1021/acs.orglett.5c02448 PMC12418491

[141] P. Arunkirirote , P. Suwalak , N. Chaisan , J. Tummatorn , S. Ruchirawat , C. Thongsornkleeb , Org. Lett. 2024, 26, 9173. 39213530 10.1021/acs.orglett.4c02708 PMC11536392

[142] I. Kim , M. Min , D. Kang , K. Kim , S. Hong , Org. Lett. 2017, 19, 1394. 28251857 10.1021/acs.orglett.7b00299

[143] A. V. Astakhov , Y. N. Tkachenko , I. V. Lavrentev , D. V. Pasyukov , M. A. Shevchenko , V. M. Chernyshev , Mendeleev Commun. 2024, 34, 238.

[144] I. Ghosh , T. Ghosh , J. I. Bardagi , B. König , Science 2014, 346, 725. 25378618 10.1126/science.1258232

[145] M. Lepori , S. Schmid , J. P. Barham , Beilstein J. Org. Chem. 2023, 19, 1055. 37533877 10.3762/bjoc.19.81 PMC10390843

[146] B. Pfund , O. S. Wenger , JACS Au 2025, 5, 426. 40017739 10.1021/jacsau.4c00974 PMC11862960

[147] C. Wang , H. Zhang , L. A. Wells , T. Liu , T. Meng , Q. Liu , P. J. Walsh , M. C. Kozlowski , T. Jia , Nat. Commun. 2021, 12, 932. 33568641 10.1038/s41467-021-21156-w PMC7876119

[148] S. Engl , O. Reiser , Chem. Soc. Rev. 2022, 51, 5287. 35703016 10.1039/d2cs00303a

[149] A. Reichle , O. Reiser , Chem. Sci. 2023, 14, 4449. 37152247 10.1039/d3sc00388d PMC10155906

[150] Y. Abderrazak , O. Reiser , ACS Catal. 2024, 14, 4847.

[151] S. Dutta , A. Mokhir , Chem. Commun. 2011, 47, 1243. 10.1039/c0cc02508a 21103531

[152] D. Bartusik , M. Minnis , G. Ghosh , A. Greer , J. Org. Chem. 2013, 78, 8537. 23899089 10.1021/jo401266r PMC3814010

[153] Y. Heo , K. Shin , M. C. Park , J. Y. Kang , Sci. Rep. 2021, 11, 5831. 33712666 10.1038/s41598-021-85107-7 PMC7954804

[154] Y. Lanchuk , A. Nikitina , N. Brezhneva , S. A. Ulasevich , S. N. Semenov , E. V. Skorb , ChemCatChem 2018, 10, 1798.

[155] R. N. Alsulami , L. Sallans , E. F. Khisamutdinov , U. Pandey , K. Glusac , R. M. Wilson , J. Photochem. Photobiol. A: Chem. 2019, 376, 224.

[156] G. M. Santiago , K. Liu , W. R. Browne , S. Otto , Nat. Chem. 2020, 12, 603. 32591744 10.1038/s41557-020-0494-4

[157] F. Liu , T. He , S. Gong , M. Shen , S. Ma , X. Huang , L. Li , L. Wang , Q. Wu , C. Gong , Acta Biomater. 2022, 154, 510. 36241016 10.1016/j.actbio.2022.10.002

[158] C.‐H. Fan , T. Xu , Z. Ke , Y.‐Y. Yeung , Org. Chem. Front. 2022, 9, 4091.

[159] T. Zhang , K. Yang , D. Liu , Y. Long , L. Chen , J. Chen , J. Phys. Chem. C 2024, 128, 13841.

